# *MaturePred*: Efficient Identification of MicroRNAs within Novel Plant Pre-miRNAs

**DOI:** 10.1371/journal.pone.0027422

**Published:** 2011-11-16

**Authors:** Ping Xuan, Maozu Guo, Yangchao Huang, Wenbin Li, Yufei Huang

**Affiliations:** 1 Department of Computer Science and Engineering, Harbin Institute of Technology, Harbin, People's Republic of China; 2 School of Computer Science and Technology, Heilongjiang University, Harbin, People's Republic of China; 3 Soybean Research Institute (Key Laboratory of Soybean Biology of Chinese Education Ministry), Northeast Agricultural University, Harbin, People's Republic of China; 4 Department of Electrical and Computer Engineering, University of Texas at San Antonio, San Antonio, Texas, United States of America; Keio University, Japan

## Abstract

**Background:**

MicroRNAs (miRNAs) are a set of short (19∼24 nt) non-coding RNAs that play significant roles as posttranscriptional regulators in animals and plants. The *ab initio* prediction methods show excellent performance for discovering new pre-miRNAs. While most of these methods can distinguish real pre-miRNAs from pseudo pre-miRNAs, few can predict the positions of miRNAs. Among the existing methods that can also predict the miRNA positions, most of them are designed for mammalian miRNAs, including human and mouse. Minority of methods can predict the positions of plant miRNAs. Accurate prediction of the miRNA positions remains a challenge, especially for plant miRNAs. This motivates us to develop *MaturePred*, a machine learning method based on support vector machine, to predict the positions of plant miRNAs for the new plant pre-miRNA candidates.

**Methodology/Principal Findings:**

A miRNA:miRNA* duplex is regarded as a whole to capture the binding characteristics of miRNAs. We extract the position-specific features, the energy related features, the structure related features, and stability related features from real/pseudo miRNA:miRNA* duplexes. A set of informative features are selected to improve the prediction accuracy. Two-stage sample selection algorithm is proposed to combat the serious imbalance problem between real and pseudo miRNA:miRNA* duplexes. The prediction method, *MaturePred*, can accurately predict plant miRNAs and achieve higher prediction accuracy compared with the existing methods. Further, we trained a prediction model with animal data to predict animal miRNAs. The model also achieves higher prediction performance. It further confirms the efficiency of our miRNA prediction method.

**Conclusions:**

The superior performance of the proposed prediction model can be attributed to the extracted features of plant miRNAs and miRNA*s, the selected training dataset, and the carefully selected features. The web service of *MaturePred*, the training datasets, the testing datasets, and the selected features are freely available at http://nclab.hit.edu.cn/maturepred/.

## Introduction

Derived from hairpin precursors (pre-miRNAs), mature microRNAs (miRNAs) are non-coding RNAs that play important roles in gene regulation by targeting mRNAs with cleavage or translational repression [1], [2]. Animal miRNAs play an important role in processes like growth processes, hematopoiesis, apoptosis, cell proliferation, and numerous diseases [3]–[5]. Plant miRNAs are involved in many important biological processes including development, metabolism, stress responses, and defense against viruses [6], [7]. In animals and plants, a primary transcript (pri-miRNA) is first cropped into the double-stranded precursor miRNA (pre-miRNA), which is further processed by Dicer or DicerLike1 (DCL1) to release the miRNA:miRNA* duplex. The stable strand of the duplex yields the mature miRNA which is incorporated into the RNA-induced silencing complex (RISC) to regulate the target mRNA.

A defining feature in miRNA biogenesis for both animals and plants is that nearly all the pre-miRNAs have the stem-loop hairpin structures. The existing of the stem loop is the key feature adopted in the *ab initio* prediction methods to distinguish real pre-miRNAs from pseudo pre-miRNAs. The machine learning algorithms have been extensively applied to learn from the real pre-miRNAs and pseudo pre-miRNAs and they include support vector machines (SVM) [8]–[12], hidden Markov model [13], [14], naïve bayes [15], random forest model [16] and kernel density estimation model [17].

Computational prediction of the positions of miRNAs can provide the most probable miRNA candidates for subsequent biological testing. Further, Plant miRNAs generally have near perfect matches to their target mRNAs. Prediction of the positions of miRNAs is helpful to identifying their target mRNAs. The function of miRNAs in regulation network can be inferred. It indicates the importance to predict the positions of miRNA candidates within the new pre-miRNAs. While the existing *ab initio* prediction methods show excellent performance for discovering new pre-miRNAs, only a few methods can predict the position of miRNAs within the new pre-miRNAs. *ProMiR* [14] implemented hidden Markov model to identify the new human pre-miRNAs. *BayesMiRNAfind* [15] used a *Naïve Bayes* classifier to predict new pre-miRNAs from mouse genome. *ProMiR* and *BayesMiRNAfind* only incorporate miRNA position prediction to increase the gene identification performance. *MatureBayes* [18] incorporated a *Naïve Bayes* classifier to identify miRNA candidates and it can accurately predict the position of miRNAs for human and mouse. *mirCos* [19] constructed a model based on SVM to predict miRNAs conserved between human and mouse. *MiRPara* [20] is designed for prediction of the miRNA candidates for animal and plant using SVM. It can predict most probable miRNA candidates from genome scale sequences. Other *ab initio* methods can only classify a pre-miRNA candidate to be real/pseudo pre-miRNA. They can not predict the position of miRNAs.

The plant pre-miRNAs usually have more complex secondary structure than the animal pre-miRNAs. Therefore, accurate prediction of the position of miRNAs within plant pre-miRNAs remains a challenge. To this end, we propose a novel prediction algorithm *MaturePred* according to the characteristics of plant pre-miRNAs. *MaturePred* regards the miRNA:miRNA* duplexes as a whole to capture more characteristics of miRNAs and miRNA*s. The new features are extracted from the real/pseudo miRNA:miRNA* duplexes. The representative pseudo miRNA:miRNA* duplexes are selected as negative training samples. An efficient model based on SVM is constructed to predict the position of miRNAs.

## Methods

### Features of plant miRNAs

Extraction of the informative features is the key for improved performance of our SVM based prediction model. The proposed model considers not only the position-specific features of a single nucleotide but also the structure-related, energy-related and stability-related features, totaling 160 features.

#### Position-specific features

The position-specific features have been defined in *MatureBayes*. Each single nucleotide is represented by one of the following 9 pairs, including the 8 possible combinations of sequence and structure and the “noValue” pair: {(*A*,*M*), (*A*,*L*), (*C*,*M*), (*C*,*L*), (*U*,*M*), (*U*,*L*), (*G*,*M*), (*G*,*L*), (*noValue*,*noValue*)}. The A (Adenie), G (Guanine), C (Cytosine), and U (Uracil) represent the nucleotide of each position, corresponding to the base composition information. M and L represent matches or mismatches of the respective nucleotide pairing. The “noValue” pair is used to indicate the lack of information on positions within the flanking region that may be located outside the limits of the pre-miRNA. The 21 position-specific features in a miRNA candidate are named as miRNA_1, miRNA_2, …, miRNA_21, respectively.

**Figure 1:**
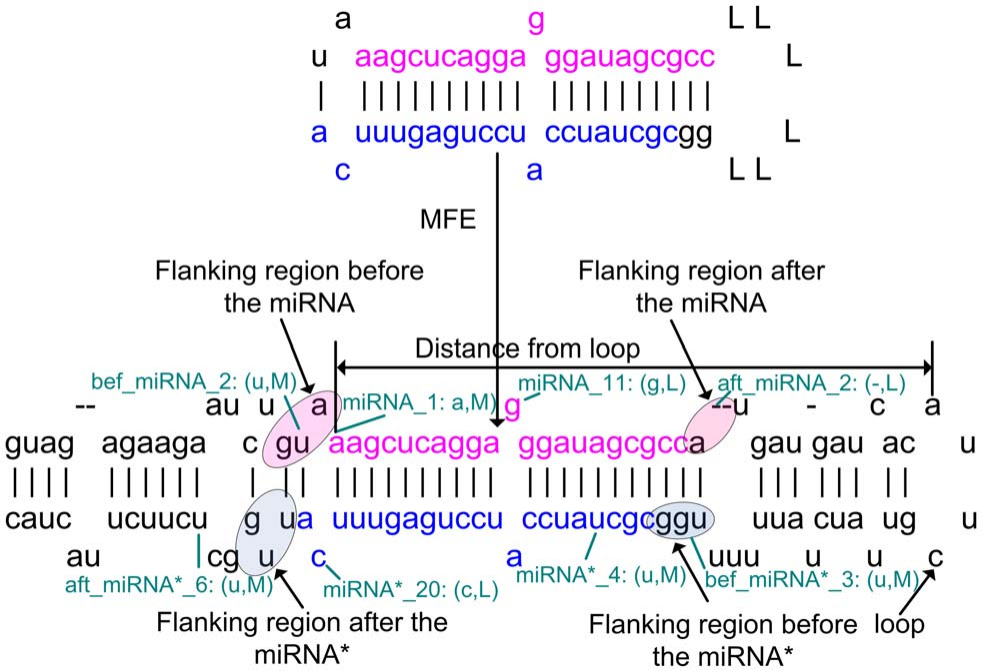
Illustration of the features used to describe the miRNA:miRNA* candidates.

As an example shown in Figure 1, the *1*-st position and *11*-th position in the miRNA are (a,M) and (g,L), respectively. The *2*-nd and the *3*-rd positions after the miRNA are “-”, representing that there is no nucleotide in the current position. This is a novel feature first proposed here and it is denoted as (-,*L*). (-,*L*) is useful for description of the position-specific information of bugles in the plant pre-miRNAs.

It is well studied that the Dicer or DCL1 usually cleaves miRNA:miRNA* duplex according to the nucleotides compositions in not only the miRNA and miRNA* but also their flanking regions [18]. Thus, the same position-specific information is also considered for the flanking regions of 12 nucleotides (nt). The 24 features in the flanking regions of a miRNA candidate are denoted as bef_miRNA_1, bef_miRNA_2, …, bef_miRNA_12, aft_miRNA_1, aft_miRNA_2, …, aft_miRNA_12. The distance of the starting position of each miRNA from the closest hairpin of the pre-miRNA is also calculated, named as *dis*.

#### New features for miRNA*

**Figure 2:**
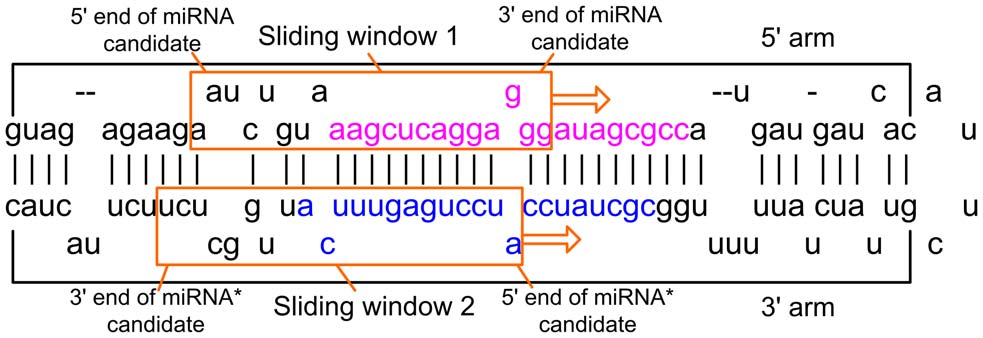
Illustration of miRNA:miRNA* candidate. This is Arabidopsis thaliana miR390a stem-loop. The 21 nucleotides in pink is the real miRNA, and the 21 nucleotides in blue is the real miRNA*.

Since the plant pre-miRNAs are cleaved into the miRNA:miRNA* duplexes, the prediction model considers the position-specific features for the whole miRNA:miRNA* duplexes. A miRNA* is defined to have the same size as the miRNA candidate but lies on the opposite strand with its 3′ end starting 2 nucleotides before the matching position of the miRNA candidate's 5′ end [1]. In order to obtain the miRNA:miRNA* candidates, two windows slide with step 1 in a pre-miRNA. As an example shown in Figure 2, if the sequence in the sliding window 1 is regarded as a miRNA candidate, the sequence in the sliding window 2 is regarded as the corresponding miRNA* candidate. The combination of window 1 and 2 is a miRNA:miRNA* candidate. When the starting position of the miRNA candidate is coincident with the starting position of the actual miRNA, the miRNA:miRNA* candidate is a real miRNA:miRNA* duplex. Otherwise, the candidate is a pseudo miRNA:miRNA* duplex.

The position-specific features are also extracted from the miRNA* candidate and its flanking regions (12 nt). The 21 position-specific features in a miRNA* candidate are named as miRNA*_1, miRNA*_2, …, miRNA*_21, respectively. The 24 features in a flanking region before/after a miRNA* candidate are denoted as bef_miRNA*_1, bef_miRNA*_2, …, bef_miRNA*_12, aft_miRNA*_1, aft_miRNA*_2, …, aft_miRNA*_12.

#### New stability-related features

According to miRNA biogenesis, the 5′ end of a miRNA is usually less stable than that of the corresponding miRNA* [6]. It is useful for determining the functional strands where the miRNAs locate. Therefore, the stability of the first nucleotide at the 5′ end of miRNA/miRNA* is considered and denoted as miRNA_5′end and miRNA*_5′end, respectively. When the first position is (A, L), (G, L), (C, L), or (U, L), the feature (miRNA_5′end/miRNA*_5′end) value is assigned to 0. When it is (G, M) or (U, M), and there is a G-U or U-G wobble pair, the feature value is assigned to 1. When it is (A, M) or (U, M), and there is an A-U or U-A pair, the feature value is assigned to 2. When it is (G, M) or (C, M), and there is a G-C or C-G pair, the feature value is assigned to 3.

#### New minimum free energy-related features

The real miRNA:miRNA* duplexes typically are of greater binding stability and are less likely to be broken. As shown in Figure 1, the miRNA candidate and the miRNA* candidate are connected by a linker sequence, “LLLLLL”. It is helpful to calculate the minimum free energy (MFE) of the miRNA:miRNA* candidate. Since “L” is not a RNA nucleotide, it does not bind with any nucleotides in the miRNA candidate and the miRNA* candidate. The MFE value of the linked miRNA candidate and miRNA* candidate is denoted as MFE_1_. In addition, the MFE value of the sequence with the flanking regions of 3 nt is calculated and denoted as MFE_2_. The one with the flanking regions of 6 nt is denoted as MFE_3_.

#### Local contiguous triplet structure features

As was defined in *triplet-SVM* [12], for any 3 adjacent nucleotides, there are 8 possible structure compositions: “(((”, “((.”, “(..”, “(.(“, “.((”, “.(.”, “..(”, and “…”. “(” and “.” represent the status of each nucleotide in the predicted secondary structure, paired or unpaired, respectively. Let *x*∈{A,C,G,U} be the middle nucleotide among the 3, and then there are 32 (4×8) possible structure-sequence combinations, which are denoted as “U(((”, “A((.”, etc. A set of these 32 triplet structure features are extracted from the miRNA candidates and the miRNA* candidates, respectively, amounting to a total of 64 triplet structure features. The 32 features from a miRNA are denoted as “miRNA_U(((”, “miRNA_A((.”, etc. and the ones from miRNA*s are denoted as “miRNA*_U(((”, “miRNA*_A((.”, etc. The triplet structure features are used to describe the miRNA candidates and miRNA* candidates in this study for the first time.

In total, 160 features are obtained from the miRNA:miRNA* candidates. The informative feature subset is selected in section *Feature Selection* to improve the prediction accuracy.

### Support vector machine

Due to the excellent generalization ability of support vector machine (SVM), we use SVM to identify real/pseudo miRNA:miRNA* duplex with *m*-dimensional (*m* = 27,48,72,136,86, see *Results and Discussion*) feature vectors. Given a training dataset *T*, each *x_i_* ∈ *T* (*i* = *1*,…,*N*) is a feature vector of real/pseudo miRNA:miRNA* duplex with the corresponding label *z_i_* (*z_i_* = +1 or −1, real miRNA:miRNA* duplex or pseudo miRNA:miRNA* duplex). SVM constructs a decision function. The decision value is used as the prediction score of the miRNA:miRNA* candidate *x*. The miRNA:miRNA* candidate with the highest prediction score for a pre-miRNA is the most probable miRNA:miRNA* duplex.
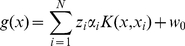
(1)
*α_i_* is the coefficient to be learned (0≤*α_i_*≤*C*) and *K* is a kernel function. In our study, a radial basis function (RBF) kernel is used, where the parameter *γ* determines the similarity level of the features so that the model becomes optimal. Since the miRNA:miRNA* duplex is considered as a whole, the kernel function is as follows.
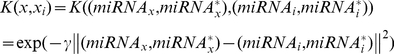
(2)


The penalty parameter *C* and the RBF kernel parameter *γ* are tuned based on the training dataset using the grid search strategy in libSVM (version 2.9).

### Construction of MaturePred with plant data

**Figure 3:**
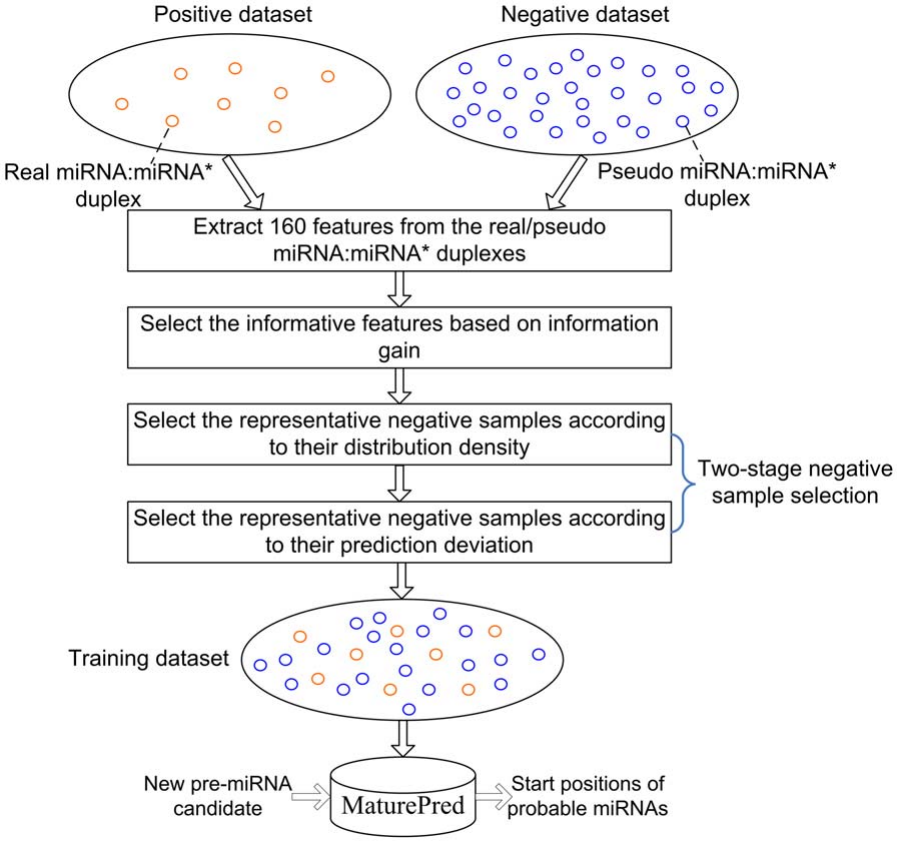
Construction of SVM prediction model based on feature selection and sample selection. Each circle represents a real/pseudo miRNA:miRNA* duplex.

A SVM based predictor called MaturePred is constructed to predict the real miRNA:miRNA* duplex and its position in a pre-miRNA. As shown in Figure 3, the process of constructing this predictor can be summarized as the following. (1) 1455 real miRNA:miRNA* duplexs from 1323 experimentally verified plant pre-miRNAs are collected as positive dataset. The 129951 pseudo miRNA:miRNA* duplexs are obtained from these pre-miRNAs as negative dataset. The 160 features are extracted from the real/pseudo miRNA:miRNA* duplexes. (2) The informative feature subset is selected through calculating the information gain of features. (3) First, the representative negative samples (pseudo miRNA:miRNA* duplexes) are selected as training samples according to their distribution density in the high-dimensional sample space. Second, the representative negative samples are selected according to their prediction deviation. (4) A SVM based plant miRNA prediction model *MaturePred* is trained by using these samples.

### Prediction of real miRNA:miRNA* duplex and the starting position

To predict the real miRNA:miRNA* duplex and its position, the secondary structure of an input pre-miRNA is first predicted by RNAfold from the Vienna package [21]. The miRNA:miRNA* candidates are then extracted from the pre-miRNAs by sliding 2 windows with step size 1 (Figure 2). *MaturePred* is applied to each of these candidates to obtain the respective prediction scores. The miRNA:miRNA* candidates are ranked by their scores and the one with highest prediction score is the most probable miRNA. The starting position of a probable miRNA is obtained as its predicted position. The feature extraction, feature selection and sample selection modules are implemented in Java. The web service of predicting the starting position of miRNAs is developed in PHP on the Linux platform.

### Prediction optimization

#### Filtering the miRNA:miRNA* candidates

**Figure 4:**
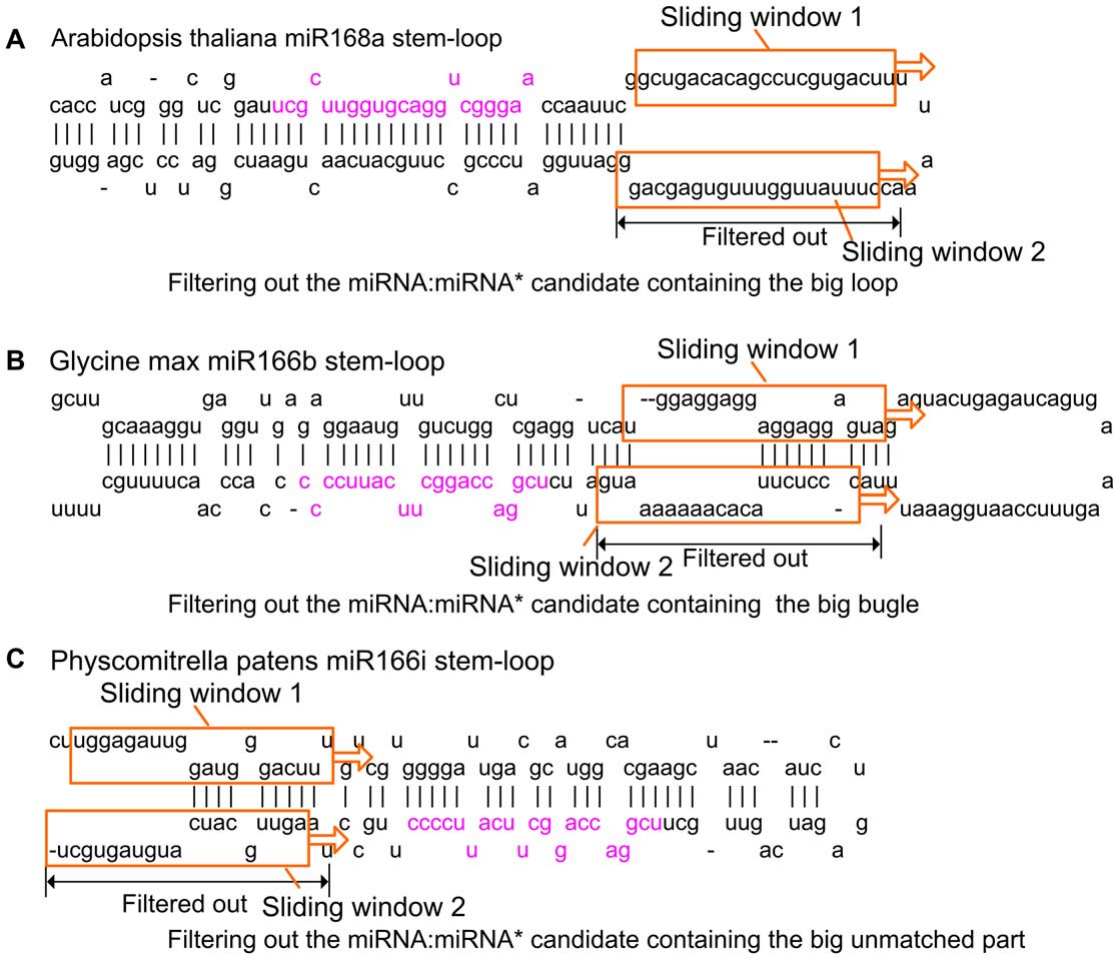
Optimizing the miRNA:miRNA* candidates. A. The candidates in the sliding windows containing the big loop are filtered out, like the one in ath-MIR168a. B. The candidates containing the big bugle are filtered out, like the one in gma-miR166b. C. The candidates containing the big unmatched part in the left end of stem are filtered out, like the one in ppt-miR166i.

The plant pre-miRNAs have more diversities than the animal pre-miRNAs. Generally, the plant pre-miRNAs have longer stems and bigger loops, as shown in Figure 4A. There could be big bugles and big unmatched regions in the stems, as shown in Figure 4B and 4C. Since the miRNAs rarely appear on the big loops, the big bugles, and the unmatched regions, the miRNA:miRNA* candidates containing them are filtered out. This filtering step can save the computational cost in the prediction process and reduce the prediction false positives.

#### Optimization of the size of sliding window and flanking region

Experimentally verified plant miRNAs from the miRBase database (version 14) [22] were collected. The minimum length, the maximum length, and the average length of these miRNAs are 19 nt, 24 nt, and 21 nt. The miRNAs of length 21 nt account for more than 60% of all plant miRNAs. Thus, the length of the sliding window is set to 21 nt. The experiment also indicated that the best prediction result is obtained when the size is 21 nt. Six different lengths of the flanking region (*s* ∈ {0,2,3,6,9,12}) were investigated by experiments. Table S1 shows that prediction performance was maximized for a flanking region of *s* = 6 nt.

### Feature selection

Feature selection aims to select a group of informative features that can retain most information of original data and lead to best prediction performance. Our adopted feature selection method considers the information gain of features.

The discrimination ability of a feature is measured by information gain based on Shannon entropy. Suppose a feature of miRNA:miRNA* duplexes is *x*, and the entropy of *x* is denoted as *H(x)*. When the value of feature *y* is given, the conditional entropy is *H(x|y)*. *IG(c,x)* is the information gain of *x* relative to the class attribute *c* [23]. *c* is assigned to 1 (real miRNA:miRNA* duplex) or −1 (pseudo miRNA:miRNA* duplex).
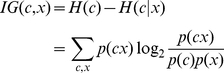
(3)


Suppose that the complete feature set is *X* = {*x_1_*, *x_2_*, …, *x_160_*}. The information gain of feature *x_i_* (1≤*i*≤160) is calculated on the dataset composed of 1455 real plant miRNA:miRNA* duplexes and 129951 pseudo plant miRNA:miRNA* duplexes. It is denoted as *IG(c*,*x_i_)*. The features with greater information gain are given higher preference.

The 160 features are categorized into 4 feature subsets: (1) position-specific feature subset *S_1_* = {miRNA_X, miRNA*_Y, bef_miRNA_Z, aft_miRNA_Z, bef_miRNA*_Z, aft_miRNA*_Z |1≤*X*,*Y*≤21, 1≤*Z*≤12} (90 features); (2) secondary structure-related feature subset *S_2_* = {“miRNA_A(((”, …, “miRNA_U…”} (32 features) and *S_3_* = {“miRNA*_A(((”, …, “miRNA*_U…”} (32 features); (3) the feature subset *S_4_* = {dis, miRNA_5′end, miRNA*_5′end, MFE_1_, MFE_2_, MFE_3_} (6 features).

In terms of *S_1_*, the feature subset evaluation indicated that the 21 position-specific features of miRNAs and that of miRNA*s are important for prediction of the starting position of miRNAs. Also, we found that the 24 features about the flanking regions (6 nt) of miRNA/miRNA* are necessary for improving the prediction accuracy (see *Feature subset evaluation*). Thus, 66 features are selected.

For each subset (*S_2_* or *S_3_*), the features are sorted by information gain in descending order. The 14 features with information gain greater than a threshold *λ* are selected. *λ* is determined by the experiments. *λ_1_* is 0.0239 for the pre-miRNAs whose miRNAs locate their 5′ arms. *λ_2_* is 0.0289 for the pre-miRNAs whose miRNAs locate their 3′ arms. In terms of *S_4_*, we found the 6 features are all important for constructing efficient prediction model. In the end, a total of 86 features are selected for plant miRNA prediction model and listed in *Feature selection result*.

### Two-stage sample selection

The plant training samples include much larger number of negative samples and the average ratio of positive samples to negative samples is nearly 1∶89. This is because the majority regions of a pre-miRNA are pseudo miRNA:miRNA* duplexes and the stems of plant pre-miRNAs are typically longer (60 nt∼more than 400 nt). It results in the serious problem of data imbalance. The prediction model constructed by such an imbalanced positive and negative dataset can only lead to poor prediction accuracy [24]. It is therefore essential to select representative negative training samples.

We proposed a two-stage sample selection algorithm. In the first stage, the density of each negative sample in its *k*-Nearest Neighbor (*k*-NN) region is estimated. The sample selection algorithm selects the representative negative samples that conform to the data distribution. In the second stage, we iteratively select the representative negative samples. The representative samples are the ones that lead to the largest deviation on the current prediction model. The negative training set is composed of the representative samples.

The *k*-NN based density estimation strategy was originally proposed to reduce data set [25]. The condensed set is effective for important data mining tasks like clustering and rule generation on large data sets. We use the *k*-NN based density estimation in the first stage.

### K-Nearest Neighbor Density Estimation

In order to calculate the distances between a negative sample (pseudo miRNA:miRNA* duplex) and its *k* neighbor samples, a distance measure is defined. Suppose that there are *m* features for each negative sample. A negative sample is represented with an *m*-dimensional feature vector. Let *v_x_* and *v_i_* be the feature vector of the *x*-th and the *i*-th negative samples, respectively. The distance between *v_x_* and *v_i_*, *d*(*v_x_*,*v_i_*), is defined by
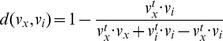
(4)where *v_x_^t^*(*v_i_^t^*) represents the transpose of vector *v_x_*(*v_i_*).

Assume that *r*_k,vi_ is the distance from *v_i_* to the *k*-th nearest negative samples. Now, let *V*(v_i_,r_k,vi_) represent the volume of the *m*-dimensional hypersphere of radius *r*_k,vi_ at *v_i_*. *g*(v_i_,r_k,vi_) is the number of negative samples in *V*(v_i_,r_k,vi_). *L* is the number of negative sample in the whole negative sample space. Then, the probability density of at *v_i_* in radius *r*_k,vi_, *f*(v_i_,r_k,vi_) can be estimated as
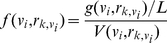
(5)


### The first stage sample selection

Suppose that the pre-miRNA data set composed of *N* pre-miRNAs, including *pre*_1_, *pre*_2_,…, and *pre*_N_. All the negative samples (pseudo miRNA:miRNA* duplexes) extracted from the *i*-th pre-miRNA *pre*_i_ are defined as the *i*-th negative sample group *G_i_*. The number of negative samples from the *i*-th pre-miRNA is *N*_i_. Since each negative sample group has its own size and distribution, the negative training samples are first selected from each negative sample group, which are merged into the overall negative training dataset *T*. The negative sample selection process of the *i*-th negative group *G_i_* is as follows.

1. For each negative sample *n_x_* ∈ *G_i_*, calculate the distance of *n_x_* from the *k*-th nearest neighbor. The distance is denoted as *r*_k,nx_. Further, the probability density of *n_x_*, *f*(n_x_,r_k,nx_), is obtained.
2. Sort the negative samples by their probability densities.
3. Select the negative sample *n_j_* ∈ *G_i_*, with the maximum *f*(n_j_,r_k,nj_) and add it into the *i*-th negative training subset *T_i_*.
4. Delete from *G_i_* all the negative samples whose the distance from *n_j_* is equal or less than *r*_k,nj._
5. Repeat steps (2)–(4) until *G_i_* is null.
6. All the negative training subset *T_i_* (1≤*i*≤N) are merged as the negative training set *T*.

**Figure 5:**
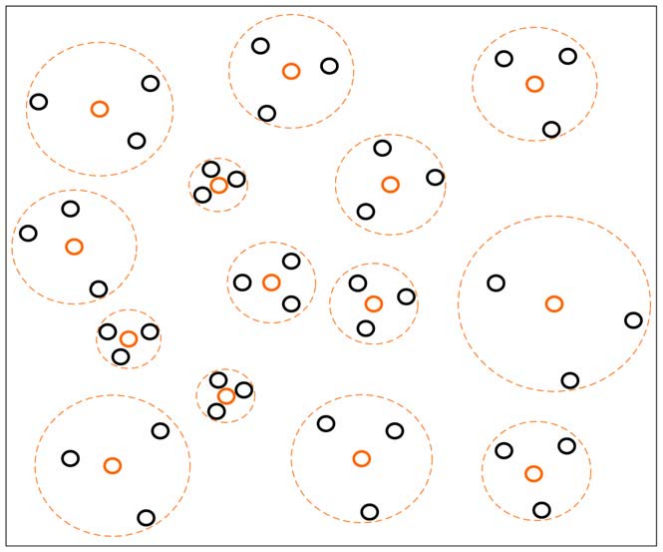
Negative sample selection based on K-NN density estimation. Each circle represents a negative sample. The circles in orange are the selected negative samples. The circles in black are the deleted samples. A big circle in dotted line represents the range covered by a selected sample.

The density based negative sample selection is illustrated in Figure 5. Since *r*_k,nj_ is inversely proportional to the estimated density at *n_j_*, regions of higher density are covered by smaller hypersphere, and sparser regions are covered by larger hypersphere. Consequently, more negative samples are selected from the regions of higher density.

The number of selected negative samples is dependent on the parameter *k*. If *k* is too great, the entire data may be represented by only a few of negative samples. Then, the selected negative samples are not sufficient to represent the entire negative sample space. If *k* is too small, the redundant negative samples will be included, which will not contribute to the improvement of the prediction performance. The *k* is determined by the prediction accuracy based on a 10-fold cross validation experiment. The *k* is chosen as 11 when the highest prediction accuracy is achieved.

### The second stage sample selection

In the second stage, the representative negative samples are iteratively collected from the remaining negative samples excluding the selected ones in the first stage. For each pre-miRNA, the positive/negative samples are selected independently. The initial training dataset *U* is composed of all the real miRNA:miRNA* duplexes (positive samples) and the selected pseudo miRNA:miRNA* duplexes (negative samples) in the first stage. The validation dataset *V* consists of all the real/pseudo miRNA:miRNA* duplexes from the *N* pre-miRNAs.

*MaturePred* is based on SVM supported by the libSVM 2.9 (http://www.csie.ntu.edu.tw/~cjlin/libsvm/). The libSVM 2.9 was changed and compiled again to get the decision value as the prediction score of a miRNA:miRNA* candidate. The candidate with the highest score is the most probable miRNA:miRNA* duplex. In the process of iteratively selecting negative samples, *MaturePred* evaluates all the positive/negative samples of in the validation set *V*. Now, let the *y*-th (1≤*y*≤4) positive sample in a pre-miRNA be denoted as *p_y_*. When the prediction is accurate, the scores of all the negative samples from the pre-miRNA are less than that of *p_y_* with the highest score. When the prediction is not sufficiently accurate, the scores of a subset of negative samples are higher than that of *p_y_* with the highest score. Let us define the prediction deviation of a miRNA:miRNA* candidate *x* as *σ*(*x*) = score(*x*)−max{score(*p_y_*)} (1≤*y*≤4). At this time, their *σ* values are more than 0. The higher the *σ* value of a negative sample is, the greater its prediction deviation is. The negative sample with the highest *σ* value is most useful for the *i*-th pre-miRNA since it causes the greatest deviation on the current prediction model.

**Figure 6:**
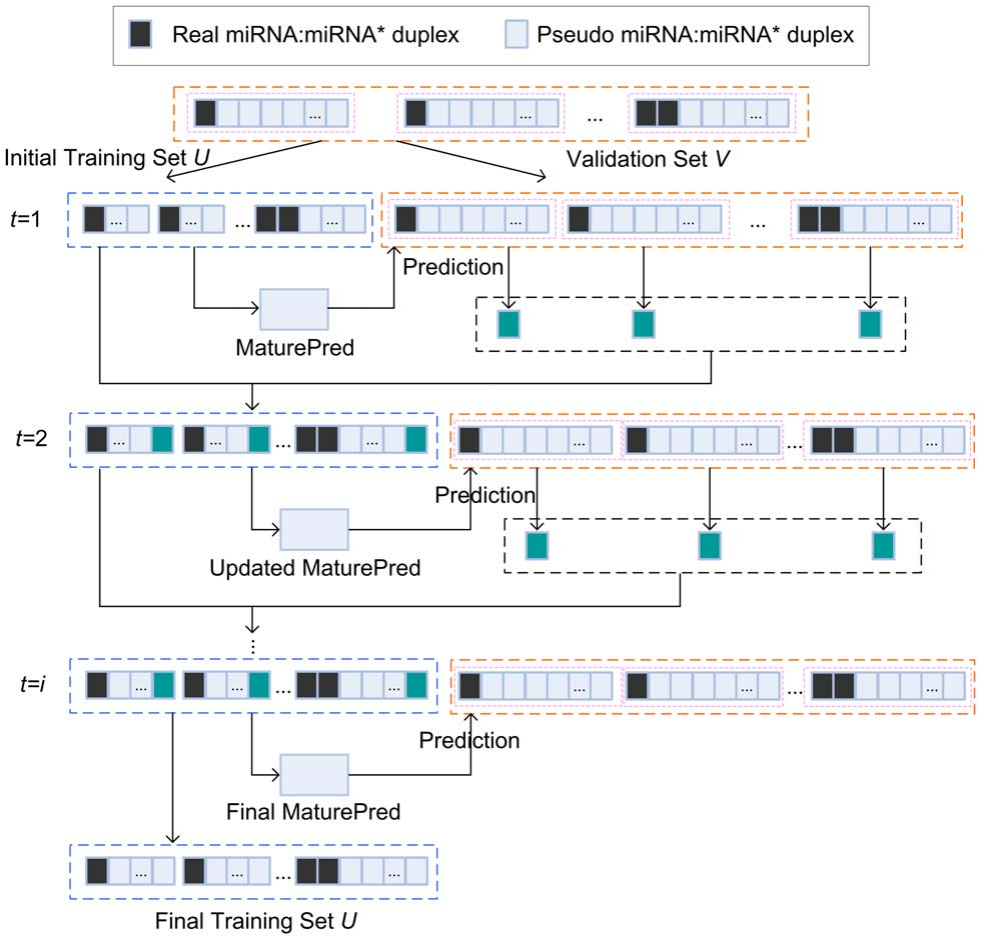
Iterative negative sample selection process.

The iterative process is demonstrated in Figure 6. The black squares represent the real miRNA:miRNA* duplexes. The grey squares represent the pseudo miRNA:miRNA* duplexes. The real and pseudo miRNA:miRNA* duplexes from a pre-miRNA are circled in pink dotted line. The iteration process of negative sample selection is as follows.

1. Initially, a prediction model *MaturePred* is constructed by the initial training dataset *U*.
2. The *MaturePred* is validated by the validation dataset *V*. The negative samples with the highest prediction deviation are selected from each pre-miRNA. They are represented by green squares in Figure 6.
3. The new selected negative samples are added into *U*. The *MaturePred* is updated with the *U*.
4. Repeat step 2–3 until all *N* pre-miRNAs satisfy termination conditions.

The iteration process will terminate the selection of negative samples for the *i*-th pre-miRNA when the predicted miRNA:miRNA* is the real miRNA:miRNA*, or all the negative samples of the *i*-th pre-miRNA are selected. When all the pre-miRNAs satisfy one of two termination conditions, the whole iteration is finished.

## Results and Discussion

### Data collection

There are 2043 plant pre-miRNAs in the miRNA database miRBase 14 (http://www.mirbase.org/), including 1366 experimentally verified pre-miRNAs. In this work, the real miRNA:miRNA* duplexes and the pseudo miRNA:miRNA* duplexes are only extracted from the experimentally verified pre-miRNAs.

#### Positive dataset

After eliminating the specific pre-miRNAs with complex secondary structures, the plant positive dataset consists of 1455 real miRNA:miRNA* duplexes from 1323 pre-miRNAs. Since some pre-miRNAs might have 2–4 miRNAs, the number of real miRNA:miRNA* duplexes is somewhat more than the number of pre-miRNAs. The real miRNA:miRNA* duplexes are extracted from the pre-miRNAs by two windows of 21 nt. The starting position of the window 1 is coincident with the starting position of the real miRNA. The combined sequence in the window 1 and 2 is a real miRNA:miRNA* duplex which is regarded as a positive sample. All the positive samples are used as the positive training samples.

#### Negative dataset

It is well known that pre-miRNAs do not produce multiple overlapping miRNAs from the same arm of the fold-back stem-loop [26]. Thus, the pseudo miRNA:miRNA* duplexes are extracted from the respective pre-miRNAs by sliding two 21 nt windows with step 1. When the starting position of the sliding window 1 does not coincide with the starting position of the real miRNA, the combined sequence in the window 1 and 2 is a pseudo miRNA:miRNA* duplex. The pseudo miRNA:miRNA* duplex is regarded as the negative sample. The plant negative dataset is composed of the 129951 negative samples from the 1323 pre-miRNAs.

#### Testing dataset

1035 experimentally verified plant pre-miRNAs have recently been reported in miRBase 15–17. These pre-miRNAs produce 1341 miRNAs. The “miR15–17 plant testing dataset” is composed of these 1341 real miRNA:miRNA* duplexes and 100807 pseudo miRNA:miRNA* duplexes. There is no overlap between the training and testing datasets as the former contains the real/pseudo miRNA:miRNA* duplexes extracted from the pre-miRNAs in miRBase 14. To assess the performance of the prediction model, the completely independent testing dataset is used.

### Evaluation method

The informative feature subset and the training samples were used to construct the prediction model *MaturePred*. The distance distribution is generated by calculating the distance between the starting position of predicted probable miRNAs and the starting position of actual miRNA. The distribution is used to evaluate the prediction performance of *MaturePred*. Assume that there are *N* pre-miRNAs in a testing dataset. For the *i*-th pre-miRNA, the position deviation between the starting position of the predicted miRNA (*p_i_*) and that of the actual miRNA (*a_i_*) is *x_i_* = *p_i_*-*a_i_*. When the predicted miRNA is in front of the actual miRNA, *x_i_* is less than 0. When the predicted miRNA is behind the actual miRNA, *x_i_* is greater than 0. The average position deviation *E(x)* is defined as
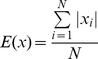
(6)


It is clear that the smaller *E(x)* is, the more accurate the position prediction is.

The strand in which a miRNA locates is referred to as the functional strand and the prediction accuracy of the functional strand is also an important criterion for assessing the prediction performance. The prediction accuracy, *P*(*y*), is defined as
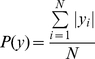
(7)where *y_i_* represents whether the predicted miRNA in the *i*-th pre-miRNA is on the functional strand. *y_i_* is assigned to 1 (on the functional strand) or 0 (not on the functional strand). The greater *P*(*y*) is, the more accurate the prediction of the functional strands is.

### Feature subset evaluation

The 160 features are extracted from the real/pseudo miRNA:miRNA* duplexes. In order to evaluate the features, they are divided into 9 subsets, including F_1_ = {21 position-specific features of miRNAs}, F_2_ = {21 position-specific features of miRNA*s}, F_3_ = {24 position-specific features of flanking regions of miRNAs}, F_4_ = {24 position-specific features of flanking regions of miRNA*s}, F_5_ = {2 stability-related features: miRNA_5′end and miRNA*_5′end}, F_6_ = {1 distance-related feature: dis}, F_7_ = {3 energy-related features: MFE_1_, MFE_2_, MFE_3_}, F_8_ = {32 structure-related features of miRNAs}, and F_9_ = {32 structure-related features of miRNA*s}. The selected feature subset has greatly effect on the prediction performance of *MaturePred*. The 4 instances of *MaturePred*: *MaturePred_27_* (27 features), *MaturePred_48_* (48 features), MaturePred*_72_* (72 features), and *MaturePred_136_* (136 features) are evaluated by performing 10-fold cross validation. With 10-fold cross validation, all real/pseudo miRNA:miRNA* duplexes in the training dataset are randomly divided into 10 equal subsets, 9 of which are used for training the prediction model, while the left out subset is used for validation. Table 1 illustrates the combination of features in each instance. “**√**” means that the whole feature subset is selected. “**△**” represents that the partial feature subset is selected. “6 nt” represents that the flanking regions are set to 6 nt long.

**Table 1:** Feature combination of MaturePred_27_∼MaturePred_86_.

Prediction model	F_1_	F_2_	F_3_	F_4_	F_5_	F_6_	F_7_	F_8_	F_9_
MaturePred_27_	**√**				**√**	**√**	**√**		
MaturePred_48_	**√**	**√**			**√**	**√**	**√**		
MaturePred_72_	**√**	**√**	6 nt	6 nt	**√**	**√**	**√**		
MaturePred_136_	**√**	**√**	**√**	**√**	**√**	**√**	**√**	**√**	**√**
MaturePred_86_	**√**	**√**	6 nt	6 nt	**√**	**√**	**√**	**△**	**△**

For each *MaturePred* instance, the representative pseudo miRNA:miRNA* duplexes are selected by the two-stage sample selection method to train the instance. We performed 10 repeated evaluations and averaged the results.

Table 2 shows the average distance between the predicted miRNAs and the actual miRNAs. *MaturePred_27_* correctly identified the functional strands for 866 of 1323 pre-miRNAs. The average position deviation is 6.273 *nt*. 43.54% of the predicted miRNAs match the starting position of actual miRNAs, while 60.26% and 85.21% are within ±2 and ±8 nt distances, respectively. Correct identification of the functional strands was successful for 976 of 1323 pre-miRNAs by *MaturePred_48_*. The average position deviation is 5.284 *nt*. 49.37% of the predicted miRNAs match the starting position of the actual miRNAs. 64.99% and 87.84% are within ±2 and ±8 nt distances, respectively. It is obviously that *MaturePred_48_* outperforms *MaturePred_27_*. *MaturePred_27_* only considered the position-specific features of miRNAs. *MaturePred_48_* considered not only the position-specific features of miRNAs but also that of miRNA*s. The prediction accuracy of functional strand (*P*) increased by 8.31%. The average position deviation (*E*) decreased by 0.989 *nt*. This indicates that it is necessary to regard the miRNA:miRNA* duplexes as a whole and consider the position-specific features of miRNAs and miRNA*s.

**Table 2:** Average distance distribution of MaturePred_27_∼MaturePred_86_.

Distance from actual miRNA	0 nt	±1 nt	±2 nt	±4 nt	±6 nt	±8 nt	*E* (nt)	*P* (%)
MaturePred_27_ (%)	43.54	54.84	60.26	70.66	78.79	85.21	6.273	65.46
MaturePred_48_ (%)	49.37	59.01	64.99	75.87	82.92	87.84	5.284	73.77
MaturePred_72_ (%)	50.66	59.95	65.44	76.37	83.64	90.45	4.889	74.45
MaturePred_136_ (%)	48.63	59.26	64.71	75.25	82.36	87.20	5.708	75.21
MaturePred_86_ (%)	51.09	61.60	67.54	77.73	85.43	90.62	4.617	74.60

It is well known that the Dicer or DCL1 usually cleaves the miRNAs according to the characteristics of the miRNAs, the miRNA*s, and their flanking regions. Thus, considering the features about the flanking regions is useful for accurate prediction of the position of miRNAs. The experimental result certificates the inference. Compared with *MaturePred_48_*, *MaturePred_72_* considered additional features of the 6 nt long flanking regions. 6 nt is the result of *Prediction optimization*. The prediction accuracy of functional strand for *MaturePred_72_* increased by 0.68%. The average position deviation decreased by 0.395 *nt*.

*MaturePred_72_* also achieved higher prediction performance than *MaturePred_136_*. It is mainly due to the 64 structure features of miRNAs and miRNA*s in *MaturePred_136_*. Since some of these features only have no or little information gain, selecting the whole 64 features would only add noise and is unfavorable to the higher prediction accuracy. It is therefore prudent to select the informative features from them.

### Feature selection result

The evaluation of different feature selections indicates that *MaturePred_72_* achieved the higher prediction accuracy. 14 informative structure-related features were selected from the 64 structure-related features (see *Feature Selection*). They are combined with the 72 features, in total 86 features. These features and the corresponding information gain are listed in Table S2. They are ranked by their normalized information gain.

The energy-related features (MFE_1_, MFE_2_, and MFE_3_) belong to the top 5 features. It shows the necessity of extracting the new energy-related features. The features about the 5′ ends of miRNAs and miRNA*s (miRNA_5′end and miRNA*_5′end) have greater information gain. These results underscore the importance of the 2 features. There are also 19 features about the miRNA*s (miRNA*_19, …, aft_miRNA*_1) ranked in the top 50 feature subset. It confirms the effectiveness of the features related to the miRNA*s. In addition, 6 of 14 triplet structure features of miRNAs and miRNA*s belong to the top 50 feature subset. It indicates the importance of these features for prediction of the position of miRNAs.

For the 21 position-specific features of miRNAs and the 12 features of flanking regions (6 nt), we found that the 1-*st*, 2-*nd*, 3-*rd*, 6-*th*, and 17–21*th* position features have greater information gain than others. In terms of miRNA*s and their flanking regions, the features of corresponding positions (19-*th*, 18-*th*, 17-*th*, 14-*th*, 1-*st*, 2-*nd*, 3-*rd*, the 1-*st* and 2-*nd* before the miRNA*s) also have greater information gain. It indicates that these position features are important for discriminating the real miRNA:miRNA* duplexes from the pseudo miRNA:miRNA* duplexes.

Table S3a shows the information gain calculated for the 711 pre-miRNAs whose miRNAs locate in their 5′ arms. S3b shows the information gain of the 744 pre-miRNAs whose miRNAs locate in their 3′ arms. S3c shows the combined information gain calculated over all pre-miRNAs in the training dataset. While the IG values of the feature *dis* in S3a and S3b are greater than those in S3c, the IG values of other features in S3a and S3b are highly consistent with the ones in S3c.

In order to validate the efficiency of the feature selection method, we tested the prediction accuracy of 86 features. As shown in Table 2, the prediction accuracy of functional strand of *MaturePred_86_* is a little worse than *MaturePred_136_*. However, *MaturePred_86_* achieved the minimum position deviation and the best distance distribution. It shows the importance of feature selection during construction of the efficient prediction model.

### Training sample selection result

In order to construct *MaturePred_86_*, 17803 representative negative samples with 86 features were selected from the negative dataset by the two-stage sample selection method. These negative samples are combined with the 1455 positive samples to form the ***selected dataset***. The existing methods including *MatureBayes* and *miRCos*, randomly selected the negative training samples. Therefore, the equal number of negative samples to the positive samples was randomly selected from the negative dataset, which are combined with the 1455 positive samples to form ***random dataset***. The ***whole dataset*** is composed of all the positive/negative samples. *MaturePred_86_* was compared with the prediction models, *MaturePred_rand_* and *MaturePred_whole_*, all of which are trained by the ***random dataset*** and the ***whole dataset*** respectively. As shown in Table 3, the miR15–17 plant testing dataset is used to evaluate the 3 prediction models.

**Table 3:** Prediction results over different training datasets.

Distance from actual miRNA	Training dataset	0 nt	±1 nt	±2 nt	±4 nt	±6 nt	±8 nt	*E* (nt)	*P* (%)
MaturePred_86_ (%)	selected dataset	35.46	46.10	54.47	64.82	73.62	78.16	5.896	68.12
MaturePred_rand_ (%)	random dataset	31.83	43.32	50.89	61.98	70.38	75.30	6.081	67.63
MaturePred_whole_ (%)	whole dataset	31.21	42.36	50.09	59.57	69.54	74.18	9.301	68.99

Although the prediction accuracy of the functional strand of *MaturePred_whole_* is a little higher than others, it obtained the worst position deviation and distance distribution. This is mainly due to the over-fitting and poor generalization of the usage of all the positive/negative samples. *MaturePred_86_* achieved higher prediction accuracy than *MaturePred_rand_*, which demonstrates that the two-stage sample selection is effective for improving the prediction accuracy. In addition, *MaturePred_rand_* achieved excellent prediction accuracy. It further confirms that the selected 86 features are sufficient to ensure the prediction performance.

### Comparison with MiRPara over plant testing data

*MiRPara* is designed for prediction of the most probable mature miRNA candidates not only for animal but also for plant. *MiRPara* is more similar to our approach as it constructed a model based on SVM. *MiRPara* and *MaturePred_86_* are evaluated by the miR15–17 plant testing dataset. The testing dataset is independent with the training dataset of *MiRPara* and that of *MaturePred*. The latest code of *MiRPara* (version of 2011-6-2) is downloaded from its website (http://159.226.126.177/mirpara/download.htm).

The SVM probability cutoff (*c*) from *MiRPara* is a threshold. When the SVM probability of a miRNA candidate is more than *c*, *MiRPara* would output the probable miRNA candidates. Here, *c* is set to 0.5. The 553 of 1035 pre-miRNAs have the probable miRNA candidates. Comparison with our method is performed on the 553 pre-miRNAs which are found to contain at least a miRNA candidate by *MiRPara*. The top 10 miRNA candidates with higher probabilities for each pre-miRNA are as the prediction result. Also, the top 10 candidates are obtained from *MaturePred_86_*. For a pre-miRNA, the distance between each one of the top 10 candidates and the actual miRNA is calculated. The minimum distance is as the prediction position deviation.

**Figure 7:**
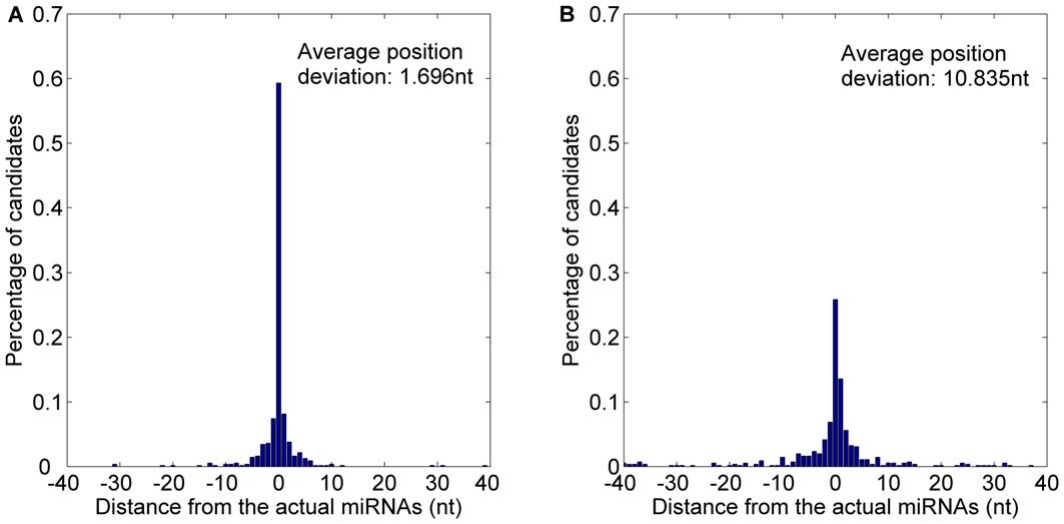
Average distance distributions of MaturePred_86_ and MiRPara over the miR15–17 plant testing dataset. A. Average distance distribution of *MaturePred_86_*. B. Average distance distribution of *MiRPara*.

The prediction result is shown in Figure 7 and detailed in Table 4. 59.31% starting position predicted by *MaturePred_86_* coincided with the respective actual miRNAs. 82.27% and 96.20% of the predicted starting position are within ±2 and ±8 nt from the actual miRNAs. The corresponding values for *MiRPara* are 25.85%, 56.05% and 76.67%. Additionally, the average position deviation (*E*) decreased by 9.139 *nt*. The result indicates that *MaturePred_86_* can give more accurate predicted miRNA candidates which are more likely to cover the actual miRNA.

**Table 4:** Prediction results of MaturePred_86_ and MiRPara over the miR15–17 plant testing dataset.

Distance from actual miRNA	0 nt	±1 nt	±2 nt	±4 nt	±6 nt	±8 nt	*E* (nt)
MaturePred_86_ (%)	59.31	74.86	82.27	91.14	95.12	96.20	1.696
MiRPara (%)	25.85	46.29	56.05	66.72	72.15	76.67	10.835

**Figure 8:**
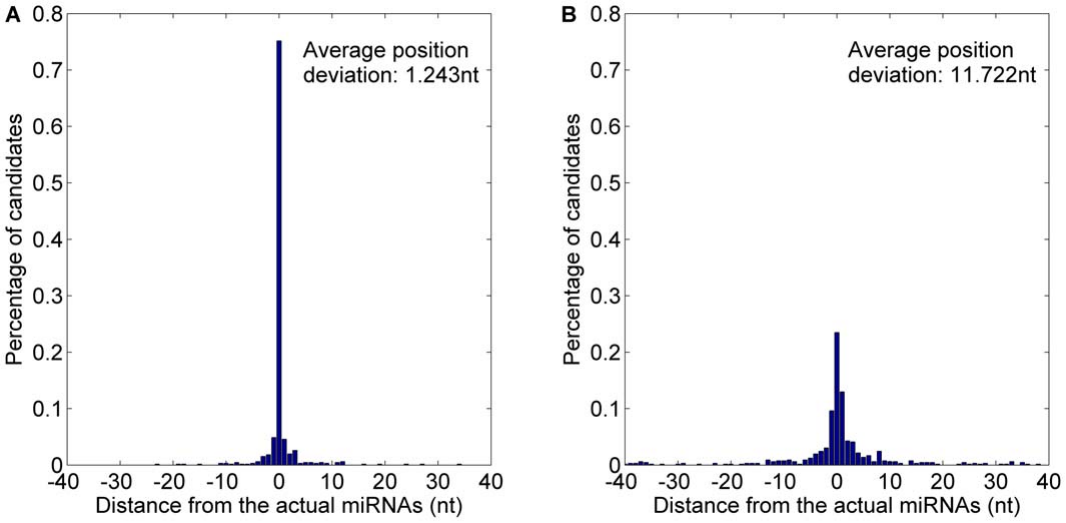
Average distance distributions of MaturePred_86_ and MiRPara over the miR13 plant testing dataset. A. Average distance distribution of *MaturePred_86_*. B. Average distance distribution of *MiRPara*.

Since both the training dataset of *MaturePred_86_* and that of *MiRPara* contain the miRNAs from the miRBase 13, these two methods are tested with these known pre-miRNAs. The parameter *c* of *MiRPara* is also set to 0.5. The 656 of 1054 pre-miRNAs have the probable miRNA candidates. The top ten prediction results of *MaturePred_86_* and *MiRPara* are compared. The detailed prediction result is shown in Table 5. The distributions of prediction distance are shown in Figure 8. 75.15% starting position predicted by *MaturePred_86_* coincided with the respective actual miRNAs. 88.41% and 96.19% of the predicted starting position are within ±2 and ±8 nt from the actual miRNAs. The corresponding values for *MiRPara* are 23.48%, 53.35% and 73.02%. Additionally, the average position deviation (*E*) decreased by 10.479 *nt*. This indicates that our method is more accurate to predict the miRNAs from the known pre-miRNAs.

**Table 5:** Prediction results of MaturePred_86_ and MiRPara over the miR13 plant testing dataset.

Distance from actual miRNA	0 nt	±1 nt	±2 nt	±4 nt	±6 nt	±8 nt	*E* (nt)
MaturePred_86_ (%)	75.15	84.60	88.41	93.45	94.82	96.19	1.243
MiRPara (%)	23.48	46.04	53.35	64.02	69.21	73.02	11.722

### Comparison with MatureBayes over plant testing data

*MatureBayes* incorporates a *Naïve Bayes* classifier to predict the starting position of miRNAs on human and mouse pre-miRNAs. Thus, *MatureBayes* has to be modified to be applicable the plant datasets since it was originally developed for human and mouse. *MatureBayes* considered totally 40 features including the 21 position-specific features of miRNAs, 18 features about the miRNA 9 nt long flanking regions, and the feature *dis*.

**Figure 9:**
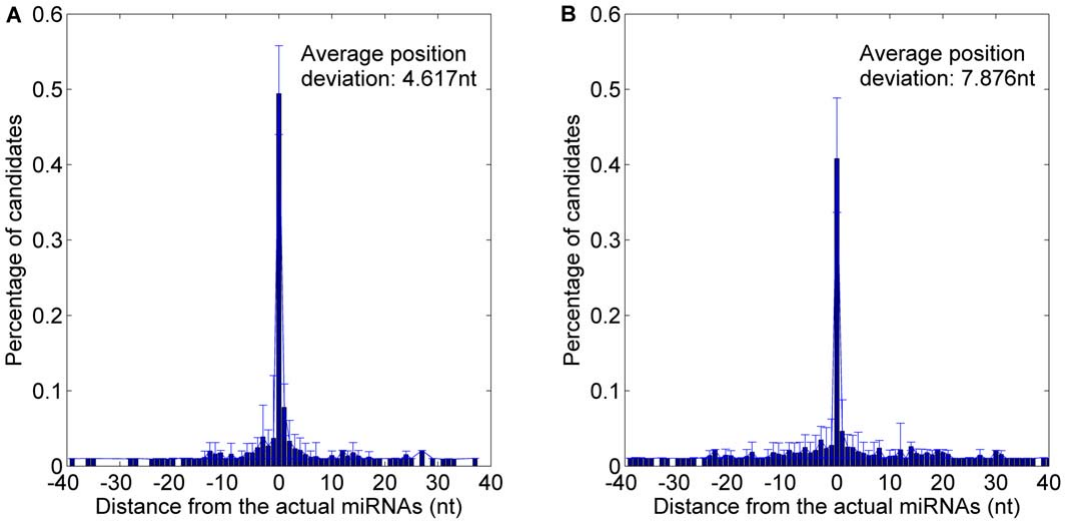
Average distance distributions over 10-fold cross validation. A. Average distance distribution of *MaturePred_86_*. B. Average distance distribution of *MatureBayes*.

*MatureBayes* offers only one the start position of the most probable miRNA candidate in any given pre-miRNA candidate. Thus, the only one is obtained from *MaturePred_86_* to compare with *MatureBayes*. *MaturePred_86_* and *MatureBayes* are evaluated by performing 10-fold cross validation. Correct identification of the functional strand(s) was successful for 987/1323 pre-miRNAs by *MaturePred_86_* versus 940/1323 pre-miRNAs by *MatureBayes*. Distance distributions between the predicted and actual miRNA starting position were calculated for each model, using the 987 and 940 pre-miRNAs, respectively. As shown in Figure 9 and detailed in Table 6, 51.09% starting position predicted by *MaturePred_86_* coincided with the respective actual miRNAs. 67.54% and 90.62% of the predicted starting position are within ±2 and ±8 nt from the actual miRNAs. The corresponding values for *MatureBayes* are 40.81%, 53.06% and 77.68%. Additionally, the prediction accuracy of functional strand (*P*) of *MaturePred_86_* increased by 3.55% and the average position deviation (*E*) decreased by 3.259 *nt*.

**Table 6:** Prediction results over different testing datasets.

Testing dataset	Size	Distance from actual miRNA	0 nt	±1 nt	±2 nt	±4 nt	±6 nt	±8 nt	*E* (nt)	*P* (%)
10-fold cross validation	1323	MaturePred_86_ (%)	51.09	61.60	67.54	77.73	85.43	90.62	4.617	74.60
		MatureBayes (%)	40.81	48.17	53.06	63.03	70.32	77.68	7.876	71.05
miR15–17 plant testing dataset	1035	MaturePred_86_ (%)	35.46	46.10	54.47	64.82	73.62	78.16	5.896	68.12
		MatureBayes (%)	27.09	33.72	38.76	47.98	54.47	59.65	10.336	67.05

**Figure 10:**
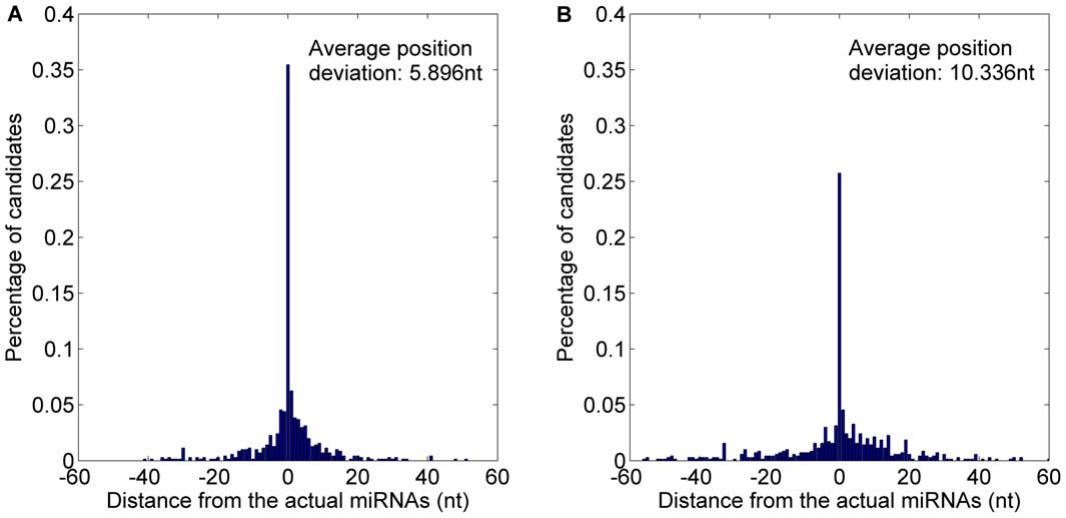
Average distance distributions over the miR15–17 plant testing dataset. A. Distance distribution of *MaturePred_86_*. B. Distance distribution of *MatureBayes*.

*MaturePred_86_* and *MatureBayes* are further evaluated by the miR15–17 plant testing dataset. This allows an unbiased analysis since the miR15–17 testing dataset was not used to build the prediction model. The functional strands of 705 pre-miRNAs were correctly identified by *MaturePred_86_* versus 694 pre-miRNAs by *MatureBayes*. As shown in Figure 10 and detailed in Table 6, the prediction accuracy of the functional strand increased in *MaturePred_86_* by 1.07% over *MatureBayes* and the average position deviation decreased by 4.44 *nt*. Taking together, we conclude that *MaturePred_86_* outperforms *MatureBayes*. The better prediction performance of *MaturePred_86_* can be attributed to the extraction of new features, the selection of the informative features, and the selection of representative negative training samples.

### Prediction of the miRNA:miRNA* duplexes

It is difficult to accurately determine the functional strands where the miRNAs locate. The experiments indicate that *MatureBayes* and *MaturePred_86_* have a similar, poor performance in terms of predicting the functional strands (around 60–70%).

**Figure 11:**
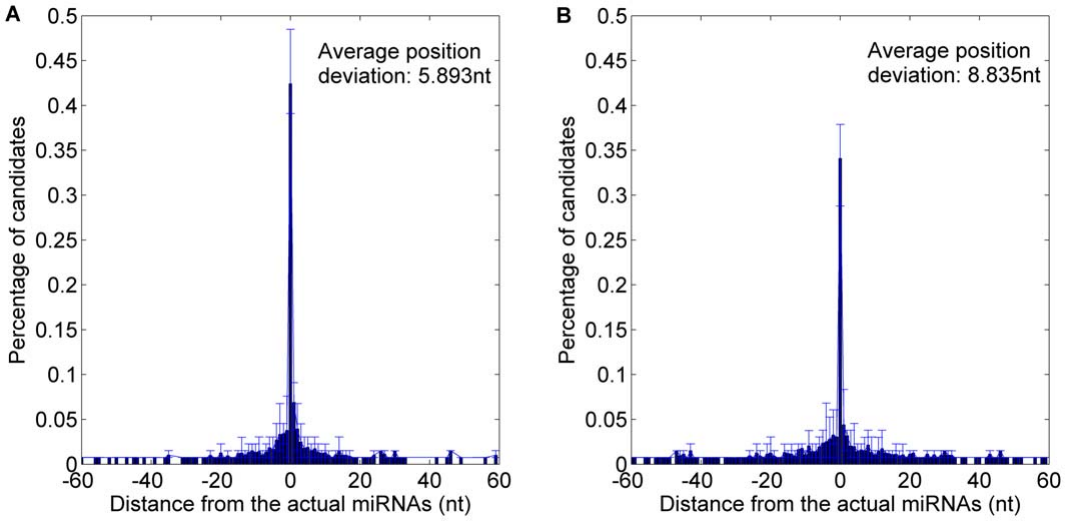
Average distance distributions over 10-fold cross validation, including 5′ arm and 3′ arm candidates. A. Average distance distribution of *MaturePred_86_*. B. Average distance distribution of *MatureBayes*.

In terms of the position prediction of human and mouse miRNAs, *MatureBayes* offers two alternatives over the 3′ arm and 5′ arm respectively to make up the inaccurate function strand prediction. We also provide the plant miRNA candidate with the highest score over the 5′ arm and the one over the 3′ arm as the more probable miRNAs. The distance between the actual miRNA(s) and the predicted candidates (locating on the same arm) were calculated. The result of 10-fold cross validation is shown in Figure 11 and detailed in Table 7. The average position deviation of *MaturePred_86_* was 2.942 *nt* less than that of *MatureBayes*.

**Table 7:** Prediction results over both arms of the pre-miRNAs.

Testing dataset	Size	Distance from actual miRNA	0 nt	±1 nt	±2 nt	±4 nt	±6 nt	±8 nt	*E* (nt)
10-fold cross validation	1323	MaturePred_86_ (%)	42.41	53.06	60.39	70.69	77.61	83.01	5.893
		MatureBayes (%)	34.09	41.49	47.92	57.22	64.54	71.04	8.835
miR15–17 plant testing dataset	1035	MaturePred_86_ (%)	30.43	42.90	51.40	63.38	72.08	77.49	6.419
		MatureBayes (%)	21.74	29.95	36.62	46.86	56.72	61.84	10.439

**Figure 12:**
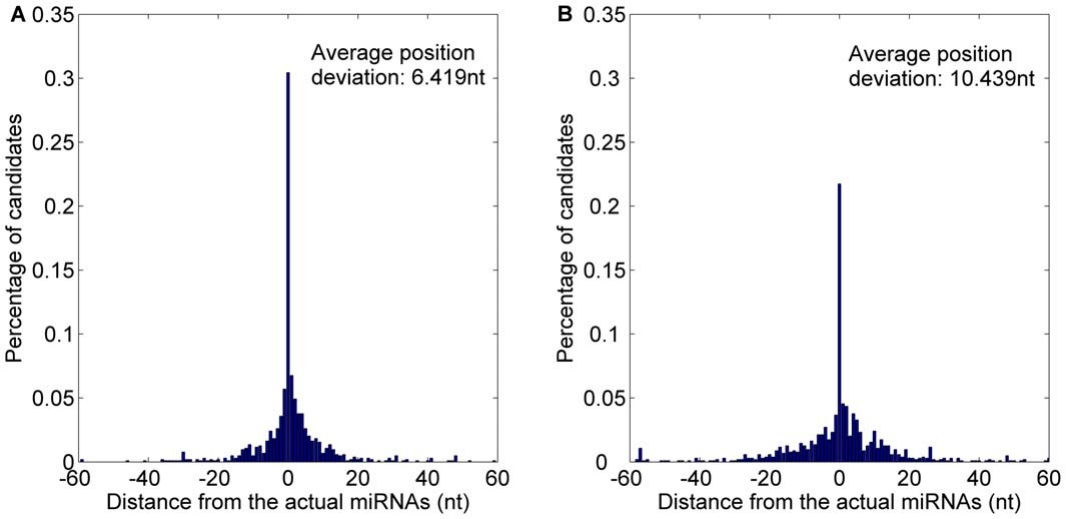
Average distance distributions over miR15–17 plant testing dataset, including 5′ arm and 3′ arm candidates. A. Distance distribution of *MaturePred_86_*. B. Distance distribution of *MatureBayes*.

In terms of the miR15–17 plant testing dataset, the average position deviation of *MaturePred_86_* decreased by 4.02 *nt*, as shown in Figure 12 and detailed in Table 7. Thus, *MaturePred_86_* outperforms *MatureBayes* in terms of giving the more probable miRNA candidates from both 5′ arms and 3′ arms.

### Construction of MaturePred with animal data

Besides constructing the prediction model for plant pre-miRNA candidates, we construct the model based on animal data for prediction of the position of miRNA in the animal pre-miRNA candidates. There are 8823 animal pre-miRNAs in the miRBase 14, including 4419 experimentally verified pre-miRNAs. 5553 real miRNA:miRNA* duplexes from the 4419 experimentally verified pre-miRNAs are collected as positive training dataset. 61866 representative pseudo miRNA:miRNA* duplexes are selected by the two stage negative sample selection algorithm as negative training dataset. The miRNAs of length 22 *nt* account for nearly 50% of all animal miRNAs. Thus, the length of the sliding window is set to 22 *nt*.

88 features are selected according to feature information gain against the animal data. These features and the corresponding information gain are listed in Table S4. Table S5 illustrates the information gain of 138 features based on animal data. As shown in Table S5, the energy-related features (MFE_1_, MFE_2_, and MFE_3_), the stability related features (miRNA_5′end, miRNA*_5′end), the partial miRNA* related features and the secondary structure related features have greater information gain. It confirms the necessity of extracting these new features again.

### Comparison with MiRPara over animal testing data

**Figure 13:**
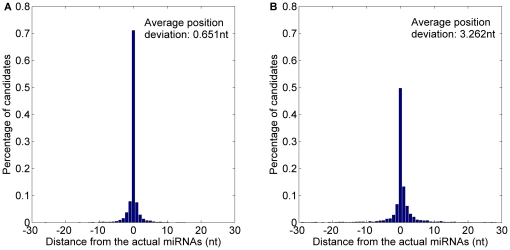
Average distance distributions of MaturePred_88_ and MiRPara over the miR15–17 animal testing dataset. A. Average distance distribution of *MaturePred_88_*. B. Average distance distribution of *MiRPara*.

The 4314 experimentally verified animal pre-miRNAs have recently been reported in miRBase 15–17. 5727 animal miRNAs from these pre-miRNAs are used to evaluate the performance of animal prediction model *MaturePred_88_* and *MiRPara*. For the *miRPara*, the 3301 of 4314 animal pre-miRNAs have the probable miRNA candidates. The top 10 probable miRNA candidates of *MaturePred_88_* and that of *MiRPara* are compared. The prediction result for the 3301 pre-miRNAs is shown in Figure 13 and Table 8. 71.07% starting position predicted by *MaturePred_88_* coincided with the respective actual miRNAs. 92.73% and 99.21% of the predicted starting position are within ±2 and ±8 nt from the actual miRNAs. The corresponding values for *MiRPara* are 49.68%, 78.46% and 91.43%. Additionally, the average position deviation (*E*) decreased by 2.611 *nt*.

**Table 8:** Prediction results of MaturePred_88_ and MiRPara over the miR15–17 animal testing dataset.

Distance from actual miRNA	0 nt	±1 nt	±2 nt	±4 nt	±6 nt	±8 nt	*E* (nt)
MaturePred_88_ (%)	71.07	86.25	92.73	97.09	98.63	99.21	0.651
MiRPara (%)	49.68	69.65	78.46	86.34	89.46	91.43	3.262

**Figure 14:**
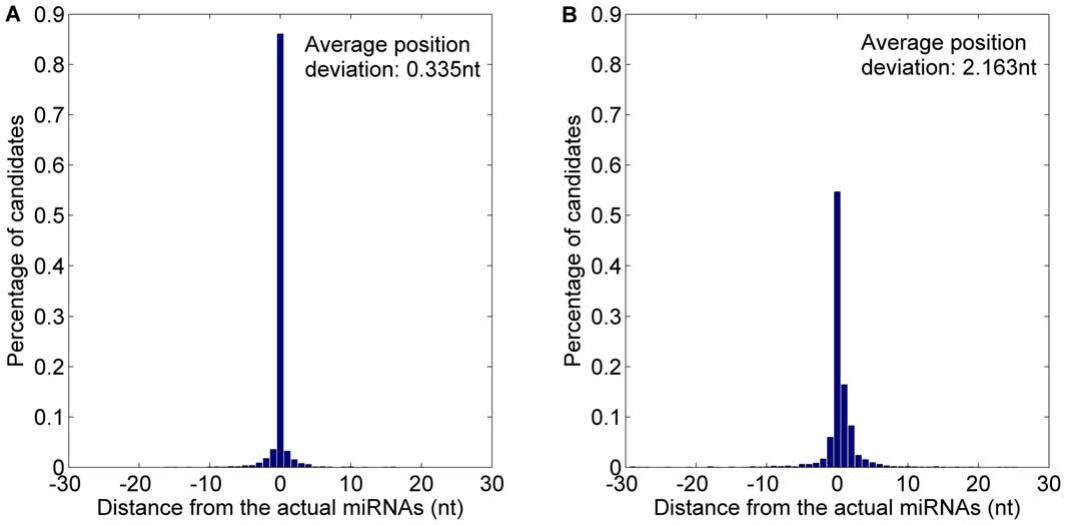
Average distance distributions of MaturePred_88_ and MiRPara over the miR13 animal testing dataset. A. Average distance distribution of *MaturePred_88_*. B. Average distance distribution of *MiRPara*.

In addition, both the training dataset of *MaturePred_88_* and that of *MiRPara* contain the miRNAs from the miRBase 13. Thus, 4985 miRNAs from 3915 experimentally verified animal pre-miRNAs are used to evaluate the performance of *MaturePred_88_* and *MiRPara* for prediction of the known miRNAs. For the *miRPara*, the 3348 of 3915 animal pre-miRNAs have the probable miRNA candidates. Figure 14 and Table 9 show the prediction results of *MaturePred_88_* and *miRPara*. 86.08% starting position predicted by *MaturePred_88_* coincided with the respective actual miRNAs. 96.05% and 99.46% of the predicted starting position are within ±2 and ±8 nt from the actual miRNAs. The corresponding values for *MiRPara* are 54.66%, 86.95% and 95.28%. The average position deviation (*E*) decreased by 1.828 *nt*. The result indicates that *MaturePred* and *MiRPara* achieve greater prediction accuracy for animal pre-miRNAs than that for plant pre-miRNAs. It is mainly due to the plant pre-miRNAs usually have more complex secondary structures than the animal pre-miRNAs.

**Table 9:** Prediction results of MaturePred_88_ and MiRPara over the miR13 animal testing dataset.

Distance from actual miRNA	0 nt	±1 nt	±2 nt	±4 nt	±6 nt	±8 nt	*E* (nt)
MaturePred_88_ (%)	86.08	92.80	96.05	98.53	99.19	99.46	0.335
MiRPara (%)	54.66	77.06	86.95	92.23	94.44	95.28	2.163

### Comparison with MatureBayes over animal testing data

Most of the existing prediction models are proposed for predicting the positions of animal miRNAs such as those of human and mouse, including *micros*, *ProMiR*, *BayesMiRNAfind* and *MatureBayes*. *MatureBayes* achieved significantly higher prediction accuracy than *ProMiR* and *BayesMiRNAfind*. Therefore, we compared *MaturePred_88_* with *MatureBayes*. *ProMiR*, *BayesMiRNAfind*, and *mirCos* can not be compared since their source code and web services are unavailable. Since *MatureBayes* mainly predicts the starting position of miRNAs on human and mouse pre-miRNAs, 927 new reported experimentally verified human and mouse pre-miRNAs in miRBase 15–17 are used to evaluate *MaturePred_88_* and *MatureBayes*. The prediction result of *MatureBayes* is obtained from its website (http://mirna.imbb.forth.gr/MatureBayes.html).

**Figure 15:**
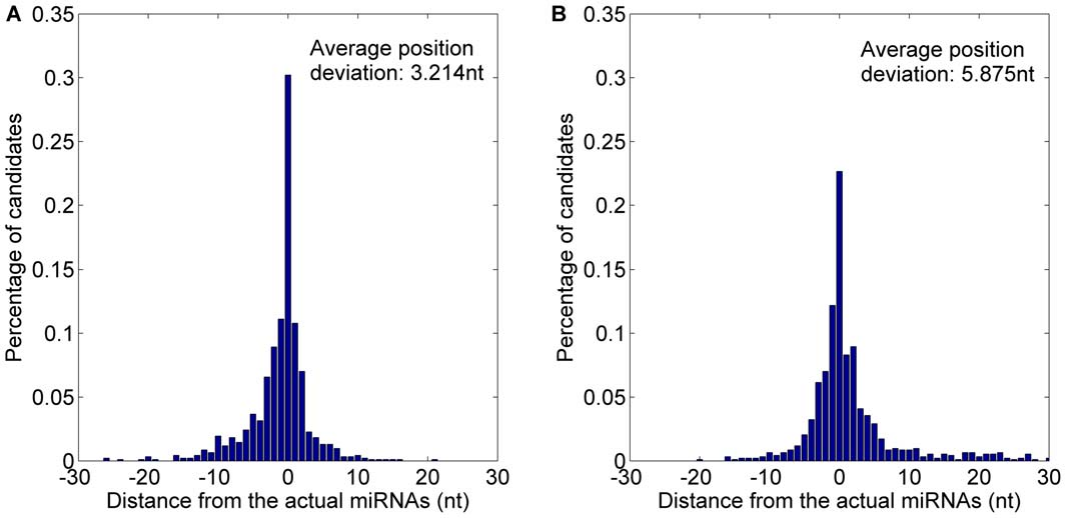
Average distance distributions of MaturePred_88_ and MatureBayes over the miR15–17 human and mouse testing dataset, including 5′ arm and 3′ arm candidates. A. Average distance distribution of *MaturePred_88_*. B. Average distance distribution of *MatureBayes*.

Since the improved *MatureBayes* offers the most probable miRNA candidates of 5′ arm and 3′ arm respectively, the ones of 5′ arm and 3′ arm are obtained from *MaturePred_88_* to compare. As shown in Figure 15 and detailed in Table 10, 30.21% starting position predicted by *MaturePred_88_* coincided with the respective actual miRNAs. 68.06% and 95.15% of the predicted starting position are within ±2 and ±8 nt from the actual miRNAs. The corresponding values for *MatureBayes* are 22.65%, 59.11% and 87.37%. The average position deviation decreased by 2.661 *nt*.

**Table 10:** Prediction results of MaturePred_88_ and MatureBayes over the miR15_17 human and mouse testing dataset.

Prediction candidates	Distance from actual miRNA	0 nt	±1 nt	±2 nt	±4 nt	±6 nt	±8 nt	*E* (nt)
Both the 5′ arm and 3′ arm candidates	MaturePred_88_ (%)	30.21	52.12	68.06	81.89	90.57	95.15	3.214
Top 10 candidates	MaturePred_88_ (%)	60.41	81.34	90.83	96.87	98.38	99.03	0.877
Both the 5′ arm and 3′ arm candidates	MatureBayes (%)	22.65	43.14	59.11	76.15	84.03	87.37	5.875

**Figure 16:**
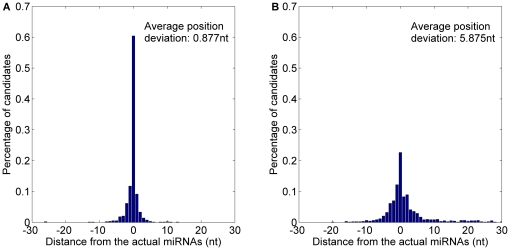
Average distance distributions of MaturePred_88_ and MatureBayes over the miR15–17 human and mouse testing dataset, including top 10 candidates. A. Average distance distribution of *MaturePred_88_*. B. Average distance distribution of *MatureBayes*.

In addition, we compared the top 10 miRNA candidates of *MaturePred_88_* with the prediction result of *MatureBayes*. As shown in Figure 16 and detailed in Table 10, 60.41% starting position predicted by *MaturePred_88_* coincided with the respective actual miRNAs. 90.83% and 99.03% of the predicted starting position are within ±2 and ±8 nt from the actual miRNAs. The average position deviation decreased by 4.998 *nt*. Specially, for the position deviations at 0 nucleotides, *MaturePred_88_* correctly identifies more than double the rate of miRNAs predicted by *MatureBayes*.

### Conclusion

A new prediction model based on SVM was developed for predicting the starting position of plant miRNAs. We demonstrated the importance of careful feature extraction, feature selection, and training sample selection in achieving effective prediction performance. Particularly, according to the characteristics of plant miRNAs, 160 features were extracted and 86 informative features were selected. Each negative sample (pseudo miRNA:miRNA* duplex) was mapped into the 86-dimensional space. 17803 representative negative samples were selected as the training samples to combat the class imbalance problem between the positive and negative samples. The proposed two-stage sample selection method can also be applied to other class imbalance problem in bioinformatics, such as identifying the SNP sites in the EST sequences.

In addition, we trained an animal miRNA prediction model with animal data. The plant model and animal model have been compared with the existing prediction methods, *MiRPara* and *MatureBayes*. The comparison results indicate that *MaturePred*, *MiRPara* and *MatureBayes* achieve higher prediction accuracy for animal pre-miRNAs than that for plant pre-miRNAs. *MaturePred* has higher prediction improvement, especially for plant pre-miRNAs. Further analysis indicated that the improvement of prediction accuracy was due to the extracted features, the selected informative features and the representative training samples. *MaturePred* can efficiently predict the positions of the more probable miRNAs in the new pre-miRNA candidates from the *ab initio* method. It can facilitate the application of the *ab initio* method in the computational prediction of miRNA genes and their function.

## Supporting Information

Table S1 **Feature combination in each prediction model, and average distance distribution of each model.**
(DOC)

Table S2 **Selected 86 features ranked by their information gain.** The features are selected over the plant dataset.
(DOC)

Table S3 **The Information gain for plant dataset.** The information gain of all 136 features for the 5′ miRNA samples, the one of all 136 features for the 3′ miRNA samples, and the one of all 136 features for the combined training dataset, including both 5′ and 3′ miRNA samples.
(DOC)

Table S4 **Selected 88 features ranked by their information gain.** The features are selected over the animal dataset.
(DOC)

Table S5 **The information gain for animal dataset.** The information gain of all 138 features for the 5′ miRNA samples, the one of all 138 features for the 3′ miRNA samples, and the one of all 138 features for the combined training dataset, including both 5′ and 3′ miRNA samples.
(DOC)
