# Supplementary material for: MaturePred: Efficient Identification of MicroRNAs within Novel Plant Pre-miRNAs
Source: PLoS One. 2011 Nov 16;6(11):e27422. doi: 10.1371/journal.pone.0027422 (PMC3217989; doi:10.1371/journal.pone.0027422)
Supplement: Table S1 — Feature combination in each prediction model, and average distance distribution of each model. (DOC) [file pone.0027422.s001.doc]

**Supplementary Table S1** Feature combination and average distance distribution of each model.

(a) Feature combination in per prediction model. F_1_={21 position-specific features of miRNAs}, F_2_={21 position-specific features of miRNA*s}, F_3_={24 position-specific features of flanking regions of miRNAs}, F_4_={24 position-specific features of flanking regions of miRNA*s}, F_5_={2 stability-related features: miRNA_5′end and miRNA*_5′end}, F_6_={1 distance-related feature: dis}, and F_7_={3 energy-related features: MFE_1_, MFE_2_, MFE_3_}. "**√**" means that the whole feature subset is selected. "*s*nt" (*s*∈{0,2,3,6,9,12}) represents that the features about the flanking regions of *s*nt are selected.

| Prediction model | No. of features | F_1_ | F_2_ | F_3_ | F_4_ | F_5_ | F_6_ | F_7_ |
| --- | --- | --- | --- | --- | --- | --- | --- | --- |
| *MaturePred_0_* | 48 | **√** | **√** | 0nt | 0nt | **√** | **√** | **√** |
| *MaturePred_2_* | 56 | **√** | **√** | 2nt | 2nt | **√** | **√** | **√** |
| *MaturePred_3_* | 60 | **√** | **√** | 3nt | 3nt | **√** | **√** | **√** |
| *MaturePred_6_* | 72 | **√** | **√** | 6nt | 6nt | **√** | **√** | **√** |
| *MaturePred_9_* | 84 | **√** | **√** | 9nt | 9nt | **√** | **√** | **√** |
| *MaturePred_12_* | 96 | **√** | **√** | 12nt | 12nt | **√** | **√** | **√** |

(b) Average distance distribution of MaturePred_0_~MaturePred_12_. *MaturePred_0_*, *MaturePred_2_*, …, *MaturePred_12_* are corresponding to the flanking region sizes (*N*∈{0,2,3,6,9,12}).

| Prediction model | No. of features | 0nt (%) | ±1nt (%) | ±2nt (%) | ±4nt (%) | ±6nt (%) | ±8nt (%) | *E* (nt) | *P* (%) |
| --- | --- | --- | --- | --- | --- | --- | --- | --- | --- |
| *MaturePred_0_* | 48 | 49.37 | 59.01 | 64.99 | 75.87 | 82.92 | 87.84 | 5.284 | 73.77 |
| *MaturePred_2_* | 56 | 50.28 | 60.15 | 64.63 | 76.88 | 83.37 | 90.25 | 4.948 | 74.30 |
| *MaturePred_3_* | 60 | 48.58 | 59.27 | 65.29 | 75.66 | 82.88 | 89.39 | 5.281 | 74.07 |
| *MaturePred_6_* | 72 | 50.66 | 59.95 | 65.44 | 76.37 | 83.64 | 90.45 | 4.889 | 74.45 |
| *MaturePred_9_* | 84 | 49.72 | 59.74 | 64.86 | 75.67 | 83.34 | 90.27 | 5.044 | 74.14 |
| *MaturePred_12_* | 96 | 47.38 | 58.04 | 63.43 | 73.90 | 81.44 | 87.72 | 5.574 | 73.17 |
