# Supplementary material for: MaturePred: Efficient Identification of MicroRNAs within Novel Plant Pre-miRNAs
Source: PLoS One. 2011 Nov 16;6(11):e27422. doi: 10.1371/journal.pone.0027422 (PMC3217989; doi:10.1371/journal.pone.0027422)
Supplement: Table S2 — Selected 86 features ranked by their information gain. The features are selected over the plant dataset. (DOC) [file pone.0027422.s002.doc]

**Supplementary Table S2** Selected 86 features ranked by their information gain.

| No. | AttrName | IG(c, attr) | No. | AttrName | IG(c, attr) | No. | AttrName | IG(c, attr) |
| --- | --- | --- | --- | --- | --- | --- | --- | --- |
| 1 | MFE_1_ | 1.0000 | 30 | miRNA_14 | 0.1698 | 59 | miRNA_12 | 0.0944 |
| 2 | miRNA_1 | 0.9547 | 31 | miRNA_16 | 0.1664 | 60 | bef_miRNA*_6 | 0.0765 |
| 3 | MFE_2_ | 0.8512 | 32 | miRNA*_6 | 0.1636 | 61 | miRNA*_11 | 0.0735 |
| 4 | miRNA*_19 | 0.8140 | 33 | miRNA*_5 | 0.1620 | 62 | aft_miRNA*_2 | 0.0728 |
| 5 | MFE_3_ | 0.5619 | 34 | miRNA*_4 | 0.1601 | 63 | bef_miRNA*_5 | 0.0726 |
| 6 | miRNA_5′end | 0.5033 | 35 | miRNA_15 | 0.1574 | 64 | aft_miRNA_3 | 0.0722 |
| 7 | miRNA_19 | 0.3611 | 36 | bef_miRNA*_3 | 0.1533 | 65 | aft_miRNA_4 | 0.0686 |
| 8 | miRNA*_1 | 0.3471 | 37 | bef_miRNA*_4 | 0.1520 | 66 | dis | 0.0649 |
| 9 | miRNA*_5′end | 0.3390 | 38 | miRNA_10 | 0.1519 | 67 | miRNA*_8 | 0.0646 |
| 10 | miRNA_3 | 0.3342 | 39 | miRNA*_15 | 0.1518 | 68 | aft_miRNA_5 | 0.0608 |
| 11 | miRNA*_17 | 0.3297 | 40 | aft_miRNA_1 | 0.1502 | 69 | aft_miRNA_6 | 0.0598 |
| 12 | miRNA_20 | 0.2813 | 41 | miRNA_C… | 0.1463 | 70 | miRNA_9 | 0.0575 |
| 13 | miRNA_21 | 0.2766 | 42 | aft_miRNA_2 | 0.1461 | 71 | bef_miRNA_4 | 0.0569 |
| 14 | miRNA_18 | 0.2653 | 43 | miRNA_5 | 0.1360 | 72 | bef_miRNA_1 | 0.0534 |
| 15 | bef_miRNA*_2 | 0.2598 | 44 | miRNA*_16 | 0.1352 | 73 | miRNA_U(.( | 0.0494 |
| 16 | bef_miRNA*_1 | 0.2556 | 45 | miRNA*_13 | 0.1326 | 74 | miRNA_11 | 0.0484 |
| 17 | miRNA*_2 | 0.2492 | 46 | aft_miRNA*_1 | 0.1250 | 75 | bef_miRNA_6 | 0.0417 |
| 18 | miRNA*_18 | 0.2462 | 47 | miRNA_G… | 0.1214 | 76 | aft_miRNA*_6 | 0.0409 |
| 19 | miRNA*_3 | 0.2230 | 48 | miRNA_7 | 0.1163 | 77 | miRNA*_9 | 0.0392 |
| 20 | miRNA_17 | 0.2131 | 49 | bef_miRNA_2 | 0.1137 | 78 | aft_miRNA*_5 | 0.0369 |
| 21 | miRNA_G((( | 0.2101 | 50 | miRNA_U… | 0.1127 | 79 | aft_miRNA*_3 | 0.0360 |
| 22 | miRNA_6 | 0.2063 | 51 | miRNA*_10 | 0.1125 | 80 | bef_miRNA_5 | 0.0356 |
| 23 | miRNA_8 | 0.2015 | 52 | miRNA_C((( | 0.1109 | 81 | aft_miRNA*_4 | 0.0334 |
| 24 | miRNA_2 | 0.1995 | 53 | bef_miRNA_3 | 0.1088 | 82 | miRNA*_20 | 0.0292 |
| 25 | miRNA*_12 | 0.1927 | 54 | miRNA_U..( | 0.1041 | 83 | miRNA*_A.(. | 0.0232 |
| 26 | miRNA_A((( | 0.1910 | 55 | miRNA*_7 | 0.1002 | 84 | miRNA*_A((( | 0.0151 |
| 27 | miRNA*_14 | 0.1889 | 56 | miRNA_13 | 0.0983 | 85 | miRNA*_A(.. | 0.0133 |
| 28 | miRNA_A… | 0.1724 | 57 | miRNA*_21 | 0.0976 | 86 | miRNA*_U..( | 0.0080 |
| 29 | miRNA_4 | 0.1708 | 58 | miRNA_A(.. | 0.0956 |  |  |  |
