# Supplementary material for: MaturePred: Efficient Identification of MicroRNAs within Novel Plant Pre-miRNAs
Source: PLoS One. 2011 Nov 16;6(11):e27422. doi: 10.1371/journal.pone.0027422 (PMC3217989; doi:10.1371/journal.pone.0027422)
Supplement: Table S3 — The Information gain for plant dataset. The information gain of all 136 features for the 5′ miRNA samples, the one of all 136 features for the 3′ miRNA samples, and the one of all 136 features for the combined training dataset, including both 5′ and 3′ miRNA samples. (DOC) [file pone.0027422.s003.doc]

**Supplementary Table S3** Information gain for plant dataset.

(a) The information gain of all 136 features for the 5′ miRNA samples

| Rank | AttrName | IG(c, attr) | Rank | AttrName | IG(c, attr) | Rank | AttrName | IG(c, attr) |
| --- | --- | --- | --- | --- | --- | --- | --- | --- |
| 1 | dis | 0.192076 | 47 | miRNA*_7 | 0.113684 | 93 | miRNA_C(.. | 0.000000 |
| 2 | bef_miRNA_6 | 0.070342 | 48 | miRNA*_8 | 0.044875 | 94 | miRNA_C… | 0.143198 |
| 3 | bef_miRNA_5 | 0.025360 | 49 | miRNA*_9 | 0.040844 | 95 | miRNA_G((( | 0.336040 |
| 4 | bef_miRNA_4 | 0.085349 | 50 | miRNA*_10 | 0.116458 | 96 | miRNA_G.(( | 0.070581 |
| 5 | bef_miRNA_3 | 0.159579 | 51 | miRNA*_11 | 0.062749 | 97 | miRNA_G(.( | 0.018244 |
| 6 | bef_miRNA_2 | 0.088766 | 52 | miRNA*_12 | 0.161934 | 98 | miRNA_G((. | 0.037590 |
| 7 | bef_miRNA_1 | 0.055567 | 53 | miRNA*_13 | 0.180931 | 99 | miRNA_G..( | 0.030579 |
| 8 | miRNA_1 | 0.824581 | 54 | miRNA*_14 | 0.189192 | 100 | miRNA_G.(. | 0.035324 |
| 9 | miRNA_2 | 0.249587 | 55 | miRNA*_15 | 0.162770 | 101 | miRNA_G(.. | 0.049251 |
| 10 | miRNA_3 | 0.335176 | 56 | miRNA*_16 | 0.117019 | 102 | miRNA_G… | 0.119062 |
| 11 | miRNA_4 | 0.163948 | 57 | miRNA*_17 | 0.331283 | 103 | miRNA*_A((( | 0.023931 |
| 12 | miRNA_5 | 0.160330 | 58 | miRNA*_18 | 0.303131 | 104 | miRNA*_A.(( | 0.019500 |
| 13 | miRNA_6 | 0.203761 | 59 | miRNA*_19 | 0.720745 | 105 | miRNA*_A(.( | 0.021214 |
| 14 | miRNA_7 | 0.154824 | 60 | miRNA*_20 | 0.046229 | 106 | miRNA*_A((. | 0.000382 |
| 15 | miRNA_8 | 0.169396 | 61 | miRNA*_21 | 0.059964 | 107 | miRNA*_A..( | 0.008175 |
| 16 | miRNA_9 | 0.054841 | 62 | aft_miRNA*_1 | 0.192683 | 108 | miRNA*_A.(. | 0.009633 |
| 17 | miRNA_10 | 0.120729 | 63 | aft_miRNA*_2 | 0.098522 | 109 | miRNA*_A(.. | 0.027143 |
| 18 | miRNA_11 | 0.037426 | 64 | aft_miRNA*_3 | 0.022019 | 110 | miRNA*_A… | 0.005754 |
| 19 | miRNA_12 | 0.084171 | 65 | aft_miRNA*_4 | 0.046769 | 111 | miRNA*_U((( | 0.017257 |
| 20 | miRNA_13 | 0.091383 | 66 | aft_miRNA*_5 | 0.058736 | 112 | miRNA*_U.(( | 0.010151 |
| 21 | miRNA_14 | 0.172581 | 67 | aft_miRNA*_6 | 0.048357 | 113 | miRNA*_U(.( | 0.016793 |
| 22 | miRNA_15 | 0.168018 | 68 | MFE_1_ | 1.000000 | 114 | miRNA*_U((. | 0.015207 |
| 23 | miRNA_16 | 0.159911 | 69 | MFE_2_ | 0.780217 | 115 | miRNA*_U..( | 0.007769 |
| 24 | miRNA_17 | 0.216874 | 70 | MFE_3_ | 0.471300 | 116 | miRNA*_U.(. | 0.005632 |
| 25 | miRNA_18 | 0.288771 | 71 | miRNA_A((( | 0.216247 | 117 | miRNA*_U(.. | 0.000186 |
| 26 | miRNA_19 | 0.335265 | 72 | miRNA_A.(( | 0.082769 | 118 | miRNA*_U… | 0.007500 |
| 27 | miRNA_20 | 0.273622 | 73 | miRNA_A(.( | 0.051146 | 119 | miRNA*_C((( | 0.018483 |
| 28 | miRNA_21 | 0.281680 | 74 | miRNA_A((. | 0.023465 | 120 | miRNA*_C.(( | 0.022225 |
| 29 | aft_miRNA_1 | 0.161702 | 75 | miRNA_A..( | 0.078141 | 121 | miRNA*_C(.( | 0.015058 |
| 30 | aft_miRNA_2 | 0.172582 | 76 | miRNA_A.(. | 0.007773 | 122 | miRNA*_C((. | 0.017061 |
| 31 | aft_miRNA_3 | 0.073638 | 77 | miRNA_A(.. | 0.077321 | 123 | miRNA*_C..( | 0.008461 |
| 32 | aft_miRNA_4 | 0.070587 | 78 | miRNA_A… | 0.150759 | 124 | miRNA*_C.(. | 0.006671 |
| 33 | aft_miRNA_5 | 0.098634 | 79 | miRNA_U((( | 0.007979 | 125 | miRNA*_C(.. | 0.004233 |
| 34 | aft_miRNA_6 | 0.086384 | 80 | miRNA_U.(( | 0.102060 | 126 | miRNA*_C… | 0.002847 |
| 35 | bef_miRNA*_6 | 0.089611 | 81 | miRNA_U(.( | 0.045299 | 127 | miRNA*_G((( | 0.018738 |
| 36 | bef_miRNA*_5 | 0.083558 | 82 | miRNA_U((. | 0.098307 | 128 | miRNA*_G.(( | 0.016575 |
| 37 | bef_miRNA*_4 | 0.200796 | 83 | miRNA_U..( | 0.150252 | 129 | miRNA*_G(.( | 0.009690 |
| 38 | bef_miRNA*_3 | 0.186719 | 84 | miRNA_U.(. | 0.004979 | 130 | miRNA*_G((. | 0.016544 |
| 39 | bef_miRNA*_2 | 0.250652 | 85 | miRNA_U(.. | 0.026290 | 131 | miRNA*_G..( | 0.007353 |
| 40 | bef_miRNA*_1 | 0.273302 | 86 | miRNA_U… | 0.126185 | 132 | miRNA*_G.(. | 0.005578 |
| 41 | miRNA*_1 | 0.357384 | 87 | miRNA_C((( | 0.209277 | 133 | miRNA*_G(.. | 0.017010 |
| 42 | miRNA*_2 | 0.271732 | 88 | miRNA_C.(( | 0.057213 | 134 | miRNA*_G… | 0.017821 |
| 43 | miRNA*_3 | 0.235086 | 89 | miRNA_C(.( | 0.048693 | 135 | miRNA_5′end | 0.435940 |
| 44 | miRNA*_4 | 0.153455 | 90 | miRNA_C((. | 0.068452 | 136 | miRNA*_5′end | 0.385581 |
| 45 | miRNA*_5 | 0.189512 | 91 | miRNA_C..( | 0.068071 |  |  |  |
| 46 | miRNA*_6 | 0.156976 | 92 | miRNA_C.(. | 0.028521 |  |  |  |

(b) The information gain of all 136 features for the 3′ miRNA samples

| Rank | AttrName | IG(c, attr) | Rank | AttrName | IG(c, attr) | Rank | AttrName | IG(c, attr) |
| --- | --- | --- | --- | --- | --- | --- | --- | --- |
| 1 | dis | 0.270238 | 47 | miRNA*_7 | 0.084173 | 93 | miRNA_C(.. | 0.006665 |
| 2 | bef_miRNA_6 | 0.040776 | 48 | miRNA*_8 | 0.095836 | 94 | miRNA_C… | 0.146205 |
| 3 | bef_miRNA_5 | 0.118260 | 49 | miRNA*_9 | 0.050086 | 95 | miRNA_G((( | 0.127867 |
| 4 | bef_miRNA_4 | 0.049071 | 50 | miRNA*_10 | 0.122883 | 96 | miRNA_G.(( | 0.023029 |
| 5 | bef_miRNA_3 | 0.110595 | 51 | miRNA*_11 | 0.093081 | 97 | miRNA_G(.( | 0.000542 |
| 6 | bef_miRNA_2 | 0.145102 | 52 | miRNA*_12 | 0.164148 | 98 | miRNA_G((. | 0.028187 |
| 7 | bef_miRNA_1 | 0.117644 | 53 | miRNA*_13 | 0.147885 | 99 | miRNA_G..( | 0.034130 |
| 8 | miRNA_1 | 0.878821 | 54 | miRNA*_14 | 0.205518 | 100 | miRNA_G.(. | 0.059180 |
| 9 | miRNA_2 | 0.245310 | 55 | miRNA*_15 | 0.200022 | 101 | miRNA_G(.. | 0.002647 |
| 10 | miRNA_3 | 0.325823 | 56 | miRNA*_16 | 0.202046 | 102 | miRNA_G… | 0.130989 |
| 11 | miRNA_4 | 0.210956 | 57 | miRNA*_17 | 0.337467 | 103 | miRNA*_A((( | 0.006815 |
| 12 | miRNA_5 | 0.165730 | 58 | miRNA*_18 | 0.286012 | 104 | miRNA*_A.(( | 0.001184 |
| 13 | miRNA_6 | 0.214178 | 59 | miRNA*_19 | 0.736538 | 105 | miRNA*_A(.( | 0.017180 |
| 14 | miRNA_7 | 0.132295 | 60 | miRNA*_20 | 0.081825 | 106 | miRNA*_A((. | 0.015151 |
| 15 | miRNA_8 | 0.165855 | 61 | miRNA*_21 | 0.141121 | 107 | miRNA*_A..( | 0.000510 |
| 16 | miRNA_9 | 0.090860 | 62 | aft_miRNA*_1 | 0.098526 | 108 | miRNA*_A.(. | 0.037192 |
| 17 | miRNA_10 | 0.151937 | 63 | aft_miRNA*_2 | 0.084998 | 109 | miRNA*_A(.. | 0.020892 |
| 18 | miRNA_11 | 0.084003 | 64 | aft_miRNA*_3 | 0.112368 | 110 | miRNA*_A… | 0.019647 |
| 19 | miRNA_12 | 0.114392 | 65 | aft_miRNA*_4 | 0.042727 | 111 | miRNA*_U((( | 0.004737 |
| 20 | miRNA_13 | 0.126124 | 66 | aft_miRNA*_5 | 0.041313 | 112 | miRNA*_U.(( | 0.004940 |
| 21 | miRNA_14 | 0.185217 | 67 | aft_miRNA*_6 | 0.051376 | 113 | miRNA*_U(.( | 0.013253 |
| 22 | miRNA_15 | 0.188331 | 68 | MFE_1_ | 1.000000 | 114 | miRNA*_U((. | 0.002727 |
| 23 | miRNA_16 | 0.195825 | 69 | MFE_2_ | 0.872099 | 115 | miRNA*_U..( | 0.028988 |
| 24 | miRNA_17 | 0.214241 | 70 | MFE_3_ | 0.609418 | 116 | miRNA*_U.(. | 0.004864 |
| 25 | miRNA_18 | 0.259594 | 71 | miRNA_A((( | 0.160784 | 117 | miRNA*_U(.. | 0.009429 |
| 26 | miRNA_19 | 0.309448 | 72 | miRNA_A.(( | 0.030691 | 118 | miRNA*_U… | 0.009074 |
| 27 | miRNA_20 | 0.265872 | 73 | miRNA_A(.( | 0.094079 | 119 | miRNA*_C((( | 0.003591 |
| 28 | miRNA_21 | 0.334120 | 74 | miRNA_A((. | 0.073484 | 120 | miRNA*_C.(( | 0.014820 |
| 29 | aft_miRNA_1 | 0.174522 | 75 | miRNA_A..( | 0.013014 | 121 | miRNA*_C(.( | 0.015321 |
| 30 | aft_miRNA_2 | 0.132732 | 76 | miRNA_A.(. | 0.023947 | 122 | miRNA*_C((. | 0.025787 |
| 31 | aft_miRNA_3 | 0.083257 | 77 | miRNA_A(.. | 0.120236 | 123 | miRNA*_C..( | 0.009328 |
| 32 | aft_miRNA_4 | 0.091506 | 78 | miRNA_A… | 0.217998 | 124 | miRNA*_C.(. | 0.002061 |
| 33 | aft_miRNA_5 | 0.046233 | 79 | miRNA_U((( | 0.036463 | 125 | miRNA*_C(.. | 0.000000 |
| 34 | aft_miRNA_6 | 0.049472 | 80 | miRNA_U.(( | 0.047432 | 126 | miRNA*_C… | 0.004024 |
| 35 | bef_miRNA*_6 | 0.092999 | 81 | miRNA_U(.( | 0.105235 | 127 | miRNA*_G((( | 0.007616 |
| 36 | bef_miRNA*_5 | 0.088714 | 82 | miRNA_U((. | 0.032170 | 128 | miRNA*_G.(( | 0.003338 |
| 37 | bef_miRNA*_4 | 0.113365 | 83 | miRNA_U..( | 0.119600 | 129 | miRNA*_G(.( | 0.005448 |
| 38 | bef_miRNA*_3 | 0.164896 | 84 | miRNA_U.(. | 0.005342 | 130 | miRNA*_G((. | 0.007669 |
| 39 | bef_miRNA*_2 | 0.333631 | 85 | miRNA_U(.. | 0.042009 | 131 | miRNA*_G..( | 0.018327 |
| 40 | bef_miRNA*_1 | 0.221468 | 86 | miRNA_U… | 0.109903 | 132 | miRNA*_G.(. | 0.012219 |
| 41 | miRNA*_1 | 0.279242 | 87 | miRNA_C((( | 0.109228 | 133 | miRNA*_G(.. | 0.006044 |
| 42 | miRNA*_2 | 0.252634 | 88 | miRNA_C.(( | 0.045800 | 134 | miRNA*_G… | 0.009009 |
| 43 | miRNA*_3 | 0.234442 | 89 | miRNA_C(.( | 0.044395 | 135 | miRNA_5′end | 0.489408 |
| 44 | miRNA*_4 | 0.198403 | 90 | miRNA_C((. | 0.076759 | 136 | miRNA*_5′end | 0.366465 |
| 45 | miRNA*_5 | 0.164366 | 91 | miRNA_C..( | 0.051527 |  |  |  |
| 46 | miRNA*_6 | 0.180559 | 92 | miRNA_C.(. | 0.010670 |  |  |  |

(c) The information gain of all 136 features for the combined training dataset, including both 5′ and 3′ miRNA samples

| Rank | AttrName | IG(c, attr) | Rank | AttrName | IG(c, attr) | Rank | AttrName | IG(c, attr) |
| --- | --- | --- | --- | --- | --- | --- | --- | --- |
| 1 | dis | 0.064946 | 47 | miRNA*_7 | 0.100198 | 93 | miRNA_C(.. | 0.002623 |
| 2 | bef_miRNA_6 | 0.041668 | 48 | miRNA*_8 | 0.064632 | 94 | miRNA_C… | 0.146295 |
| 3 | bef_miRNA_5 | 0.035611 | 49 | miRNA*_9 | 0.039237 | 95 | miRNA_G((( | 0.210086 |
| 4 | bef_miRNA_4 | 0.056900 | 50 | miRNA*_10 | 0.112525 | 96 | miRNA_G.(( | 0.011329 |
| 5 | bef_miRNA_3 | 0.108828 | 51 | miRNA*_11 | 0.073502 | 97 | miRNA_G(.( | 0.000598 |
| 6 | bef_miRNA_2 | 0.113693 | 52 | miRNA*_12 | 0.192689 | 98 | miRNA_G((. | 0.006582 |
| 7 | bef_miRNA_1 | 0.053381 | 53 | miRNA*_13 | 0.132593 | 99 | miRNA_G..( | 0.022385 |
| 8 | miRNA_1 | 0.954696 | 54 | miRNA*_14 | 0.188866 | 100 | miRNA_G.(. | 0.046684 |
| 9 | miRNA_2 | 0.199500 | 55 | miRNA*_15 | 0.151823 | 101 | miRNA_G(.. | 0.010636 |
| 10 | miRNA_3 | 0.334188 | 56 | miRNA*_16 | 0.135248 | 102 | miRNA_G… | 0.121429 |
| 11 | miRNA_4 | 0.170758 | 57 | miRNA*_17 | 0.329737 | 103 | miRNA*_A((( | 0.015106 |
| 12 | miRNA_5 | 0.136026 | 58 | miRNA*_18 | 0.246161 | 104 | miRNA*_A.(( | 0.000942 |
| 13 | miRNA_6 | 0.206256 | 59 | miRNA*_19 | 0.814012 | 105 | miRNA*_A(.( | 0.013006 |
| 14 | miRNA_7 | 0.116336 | 60 | miRNA*_20 | 0.029193 | 106 | miRNA*_A((. | 0.001805 |
| 15 | miRNA_8 | 0.201537 | 61 | miRNA*_21 | 0.097581 | 107 | miRNA*_A..( | 0.000297 |
| 16 | miRNA_9 | 0.057467 | 62 | aft_miRNA*_1 | 0.124951 | 108 | miRNA*_A.(. | 0.023168 |
| 17 | miRNA_10 | 0.151904 | 63 | aft_miRNA*_2 | 0.072816 | 109 | miRNA*_A(.. | 0.013327 |
| 18 | miRNA_11 | 0.048405 | 64 | aft_miRNA*_3 | 0.035962 | 110 | miRNA*_A… | 0.010166 |
| 19 | miRNA_12 | 0.094412 | 65 | aft_miRNA*_4 | 0.033378 | 111 | miRNA*_U((( | 0.001619 |
| 20 | miRNA_13 | 0.098253 | 66 | aft_miRNA*_5 | 0.036886 | 112 | miRNA*_U.(( | 0.006584 |
| 21 | miRNA_14 | 0.169821 | 67 | aft_miRNA*_6 | 0.040855 | 113 | miRNA*_U(.( | 0.017092 |
| 22 | miRNA_15 | 0.157357 | 68 | MFE_1_ | 1.000000 | 114 | miRNA*_U((. | 0.000000 |
| 23 | miRNA_16 | 0.166371 | 69 | MFE_2_ | 0.851237 | 115 | miRNA*_U..( | 0.008040 |
| 24 | miRNA_17 | 0.213135 | 70 | MFE_3_ | 0.561858 | 116 | miRNA*_U.(. | 0.001534 |
| 25 | miRNA_18 | 0.265301 | 71 | miRNA_A((( | 0.190975 | 117 | miRNA*_U(.. | 0.003585 |
| 26 | miRNA_19 | 0.361092 | 72 | miRNA_A.(( | 0.006470 | 118 | miRNA*_U… | 0.007051 |
| 27 | miRNA_20 | 0.281321 | 73 | miRNA_A(.( | 0.066007 | 119 | miRNA*_C((( | 0.001055 |
| 28 | miRNA_21 | 0.276551 | 74 | miRNA_A((. | 0.025129 | 120 | miRNA*_C.(( | 0.015491 |
| 29 | aft_miRNA_1 | 0.150192 | 75 | miRNA_A..( | 0.012122 | 121 | miRNA*_C(.( | 0.008607 |
| 30 | aft_miRNA_2 | 0.146118 | 76 | miRNA_A.(. | 0.015836 | 122 | miRNA*_C((. | 0.016144 |
| 31 | aft_miRNA_3 | 0.072201 | 77 | miRNA_A(.. | 0.095620 | 123 | miRNA*_C..( | 0.005199 |
| 32 | aft_miRNA_4 | 0.068580 | 78 | miRNA_A… | 0.172383 | 124 | miRNA*_C.(. | 0.003238 |
| 33 | aft_miRNA_5 | 0.060842 | 79 | miRNA_U((( | 0.011378 | 125 | miRNA*_C(.. | 0.001890 |
| 34 | aft_miRNA_6 | 0.059754 | 80 | miRNA_U.(( | 0.054349 | 126 | miRNA*_C… | 0.003406 |
| 35 | bef_miRNA*_6 | 0.076462 | 81 | miRNA_U(.( | 0.049378 | 127 | miRNA*_G((( | 0.013026 |
| 36 | bef_miRNA*_5 | 0.072556 | 82 | miRNA_U((. | 0.042050 | 128 | miRNA*_G.(( | 0.002661 |
| 37 | bef_miRNA*_4 | 0.151994 | 83 | miRNA_U..( | 0.104136 | 129 | miRNA*_G(.( | 0.003555 |
| 38 | bef_miRNA*_3 | 0.153322 | 84 | miRNA_U.(. | 0.003798 | 130 | miRNA*_G((. | 0.001936 |
| 39 | bef_miRNA*_2 | 0.259784 | 85 | miRNA_U(.. | 0.032853 | 131 | miRNA*_G..( | 0.005222 |
| 40 | bef_miRNA*_1 | 0.255571 | 86 | miRNA_U… | 0.112675 | 132 | miRNA*_G.(. | 0.009101 |
| 41 | miRNA*_1 | 0.347104 | 87 | miRNA_C((( | 0.110884 | 133 | miRNA*_G(.. | 0.003063 |
| 42 | miRNA*_2 | 0.249206 | 88 | miRNA_C.(( | 0.041906 | 134 | miRNA*_G… | 0.005792 |
| 43 | miRNA*_3 | 0.223034 | 89 | miRNA_C(.( | 0.030201 | 135 | miRNA_5′end | 0.503287 |
| 44 | miRNA*_4 | 0.160123 | 90 | miRNA_C((. | 0.059167 | 136 | miRNA*_5′end | 0.338976 |
| 45 | miRNA*_5 | 0.162037 | 91 | miRNA_C..( | 0.054969 |  |  |  |
| 46 | miRNA*_6 | 0.163611 | 92 | miRNA_C.(. | 0.012844 |  |  |  |
