# Supplementary material for: MaturePred: Efficient Identification of MicroRNAs within Novel Plant Pre-miRNAs
Source: PLoS One. 2011 Nov 16;6(11):e27422. doi: 10.1371/journal.pone.0027422 (PMC3217989; doi:10.1371/journal.pone.0027422)
Supplement: Table S4 — Selected 88 features ranked by their information gain. The features are selected over the animal dataset. (DOC) [file pone.0027422.s004.doc]

**Supplementary Table S4** Selected 88 features ranked by their information gain.

| No. | AttrName | IG(c, attr) | No. | AttrName | IG(c, attr) | No. | AttrName | IG(c, attr) |
| --- | --- | --- | --- | --- | --- | --- | --- | --- |
| 1 | dis | 1 | 31 | bef_miRNA_1 | 0.1333 | 61 | miRNA_8 | 0.0701 |
| 2 | miRNA_1 | 0.7892 | 32 | miRNA*_14 | 0.1295 | 62 | miRNA_9 | 0.0688 |
| 3 | MFE_1_ | 0.5775 | 33 | miRNA*_19 | 0.1281 | 63 | bef_miRNA_3 | 0.0676 |
| 4 | MFE_2_ | 0.444 | 34 | miRNA_7 | 0.1234 | 64 | aft_miRNA*_3 | 0.0633 |
| 5 | miRNA_21 | 0.2947 | 35 | miRNA*_5 | 0.1225 | 65 | miRNA*_12 | 0.0626 |
| 6 | miRNA_22 | 0.2935 | 36 | miRNA*_15 | 0.1211 | 66 | miRNA_13 | 0.0618 |
| 7 | bef_miRNA*_1 | 0.2931 | 37 | miRNA*_21 | 0.1206 | 67 | bef_miRNA*_6 | 0.0538 |
| 8 | bef_miRNA*_2 | 0.2776 | 38 | miRNA*_22 | 0.12 | 68 | miRNA_A(.( | 0.0492 |
| 9 | miRNA*_20 | 0.2724 | 39 | miRNA_U((( | 0.1185 | 69 | miRNA*_8 | 0.0474 |
| 10 | MFE_3_ | 0.2566 | 40 | aft_miRNA_3 | 0.1181 | 70 | miRNA_U..( | 0.0464 |
| 11 | miRNA_5'end | 0.2458 | 41 | miRNA*_17 | 0.1155 | 71 | miRNA_G… | 0.0463 |
| 12 | miRNA*_1 | 0.22 | 42 | bef_miRNA*_4 | 0.1116 | 72 | aft_miRNA_4 | 0.0453 |
| 13 | aft_miRNA*_6 | 0.2093 | 43 | miRNA_14 | 0.1098 | 73 | aft_miRNA*_2 | 0.0452 |
| 14 | miRNA_20 | 0.2083 | 44 | bef_miRNA_2 | 0.1083 | 74 | miRNA_C(.. | 0.0442 |
| 15 | aft_miRNA_1 | 0.2002 | 45 | miRNA_15 | 0.1046 | 75 | miRNA_A(.. | 0.0397 |
| 16 | miRNA*_5'end | 0.1837 | 46 | miRNA*_16 | 0.1029 | 76 | miRNA*_9 | 0.0369 |
| 17 | miRNA_19 | 0.1819 | 47 | bef_miRNA*_5 | 0.1007 | 77 | bef_miRNA_5 | 0.0361 |
| 18 | bef_miRNA*_3 | 0.1799 | 48 | miRNA*_6 | 0.0985 | 78 | miRNA*_11 | 0.0359 |
| 19 | aft_miRNA*_5 | 0.1776 | 49 | miRNA_5 | 0.0957 | 79 | bef_miRNA_4 | 0.0347 |
| 20 | miRNA*_2 | 0.1744 | 50 | miRNA*_7 | 0.0936 | 80 | miRNA_11 | 0.0302 |
| 21 | miRNA_18 | 0.1708 | 51 | miRNA_A((( | 0.0889 | 81 | miRNA_C..( | 0.0302 |
| 22 | miRNA_17 | 0.1685 | 52 | bef_miRNA_6 | 0.0871 | 82 | miRNA_A… | 0.0297 |
| 23 | miRNA*_4 | 0.1622 | 53 | miRNA_6 | 0.0865 | 83 | miRNA_C((( | 0.0281 |
| 24 | miRNA*_3 | 0.1586 | 54 | miRNA_U… | 0.0799 | 84 | miRNA*_10 | 0.0276 |
| 25 | miRNA_G((( | 0.1515 | 55 | miRNA_C… | 0.0797 | 85 | miRNA_C(.( | 0.0254 |
| 26 | miRNA_4 | 0.1503 | 56 | miRNA*_13 | 0.0795 | 86 | aft_miRNA_6 | 0.0251 |
| 27 | miRNA_3 | 0.1476 | 57 | miRNA*_18 | 0.0779 | 87 | aft_miRNA_5 | 0.0203 |
| 28 | aft_miRNA_2 | 0.1393 | 58 | aft_miRNA*_1 | 0.0748 | 88 | miRNA_10 | 0.0159 |
| 29 | miRNA_16 | 0.1382 | 59 | miRNA_12 | 0.0737 |  |  |  |
| 30 | miRNA_2 | 0.1377 | 60 | aft_miRNA*_4 | 0.0729 |  |  |  |
