# Supplementary material for: MaturePred: Efficient Identification of MicroRNAs within Novel Plant Pre-miRNAs
Source: PLoS One. 2011 Nov 16;6(11):e27422. doi: 10.1371/journal.pone.0027422 (PMC3217989; doi:10.1371/journal.pone.0027422)
Supplement: Table S5 — The information gain for animal dataset. The information gain of all 138 features for the 5′ miRNA samples, the one of all 138 features for the 3′ miRNA samples, and the one of all 138 features for the combined training dataset, including both 5′ and 3′ miRNA samples. (DOC) [file pone.0027422.s005.doc]

**Supplementary Table S5** Information gain for animal dataset.

(a) The information gain of all 138 features for the 5′ miRNA samples

| Rank | AttrName | IG(c, attr) | Rank | AttrName | IG(c, attr) | Rank | AttrName | IG(c, attr) |
| --- | --- | --- | --- | --- | --- | --- | --- | --- |
| 1 | dis | 1.000000 | 47 | miRNA*_6 | 0.098483 | 93 | miRNA_C..( | 0.030673 |
| 2 | bef_miRNA_6 | 0.071271 | 48 | miRNA*_7 | 0.090021 | 94 | miRNA_C.(. | 0.010698 |
| 3 | bef_miRNA_5 | 0.058641 | 49 | miRNA*_8 | 0.025457 | 95 | miRNA_C(.. | 0.048990 |
| 4 | bef_miRNA_4 | 0.064490 | 50 | miRNA*_9 | 0.034676 | 96 | miRNA_C… | 0.080080 |
| 5 | bef_miRNA_3 | 0.043803 | 51 | miRNA*_10 | 0.037144 | 97 | miRNA_G((( | 0.161897 |
| 6 | bef_miRNA_2 | 0.091232 | 52 | miRNA*_11 | 0.040395 | 98 | miRNA_G.(( | 0.055572 |
| 7 | bef_miRNA_1 | 0.097116 | 53 | miRNA*_12 | 0.076556 | 99 | miRNA_G(.( | 0.015962 |
| 8 | miRNA_1 | 0.722764 | 54 | miRNA*_13 | 0.105126 | 100 | miRNA_G((. | 0.000188 |
| 9 | miRNA_2 | 0.118234 | 55 | miRNA*_14 | 0.177231 | 101 | miRNA_G..( | 0.022473 |
| 10 | miRNA_3 | 0.114316 | 56 | miRNA*_15 | 0.161453 | 102 | miRNA_G.(. | 0.019142 |
| 11 | miRNA_4 | 0.170564 | 57 | miRNA*_16 | 0.117924 | 103 | miRNA_G(.. | 0.008664 |
| 12 | miRNA_5 | 0.111890 | 58 | miRNA*_17 | 0.154380 | 104 | miRNA_G… | 0.040340 |
| 13 | miRNA_6 | 0.133570 | 59 | miRNA*_18 | 0.081544 | 105 | miRNA*_A((( | 0.009095 |
| 14 | miRNA_7 | 0.176508 | 60 | miRNA*_19 | 0.115041 | 106 | miRNA*_A.(( | 0.001204 |
| 15 | miRNA_8 | 0.088283 | 61 | miRNA*_20 | 0.234224 | 107 | miRNA*_A(.( | 0.001624 |
| 16 | miRNA_9 | 0.070676 | 62 | miRNA*_21 | 0.074608 | 108 | miRNA*_A((. | 0.001900 |
| 17 | miRNA_10 | 0.022730 | 63 | miRNA*_22 | 0.097282 | 109 | miRNA*_A..( | 0.005431 |
| 18 | miRNA_11 | 0.023469 | 64 | aft_miRNA*_1 | 0.031452 | 110 | miRNA*_A.(. | 0.002403 |
| 19 | miRNA_12 | 0.070324 | 65 | aft_miRNA*_2 | 0.042133 | 111 | miRNA*_A(.. | 0.009504 |
| 20 | miRNA_13 | 0.042671 | 66 | aft_miRNA*_3 | 0.073066 | 112 | miRNA*_A… | 0.020595 |
| 21 | miRNA_14 | 0.100529 | 67 | aft_miRNA*_4 | 0.040033 | 113 | miRNA*_U((( | 0.014046 |
| 22 | miRNA_15 | 0.105468 | 68 | aft_miRNA*_5 | 0.097707 | 114 | miRNA*_U.(( | 0.001388 |
| 23 | miRNA_16 | 0.126386 | 69 | aft_miRNA*_6 | 0.063078 | 115 | miRNA*_U(.( | 0.003060 |
| 24 | miRNA_17 | 0.169508 | 70 | MFE1 | 0.648660 | 116 | miRNA*_U((. | 0.000680 |
| 25 | miRNA_18 | 0.162275 | 71 | MFE2 | 0.429666 | 117 | miRNA*_U..( | 0.002983 |
| 26 | miRNA_19 | 0.180937 | 72 | MFE3 | 0.271305 | 118 | miRNA*_U.(. | 0.000393 |
| 27 | miRNA_20 | 0.193541 | 73 | miRNA_A((( | 0.132047 | 119 | miRNA*_U(.. | 0.007170 |
| 28 | miRNA_21 | 0.287528 | 74 | miRNA_A.(( | 0.009129 | 120 | miRNA*_U… | 0.012034 |
| 29 | miRNA_22 | 0.290301 | 75 | miRNA_A(.( | 0.047868 | 121 | miRNA*_C((( | 0.004687 |
| 30 | aft_miRNA_1 | 0.170221 | 76 | miRNA_A((. | 0.001854 | 122 | miRNA*_C.(( | 0.001574 |
| 31 | aft_miRNA_2 | 0.129929 | 77 | miRNA_A..( | 0.045905 | 123 | miRNA*_C(.( | 0.003007 |
| 32 | aft_miRNA_3 | 0.116782 | 78 | miRNA_A.(. | 0.003128 | 124 | miRNA*_C((. | 0.009987 |
| 33 | aft_miRNA_4 | 0.045539 | 79 | miRNA_A(.. | 0.050003 | 125 | miRNA*_C..( | 0.006186 |
| 34 | aft_miRNA_5 | 0.026942 | 80 | miRNA_A… | 0.110655 | 126 | miRNA*_C.(. | 0.007288 |
| 35 | aft_miRNA_6 | 0.071741 | 81 | miRNA_U((( | 0.137140 | 127 | miRNA*_C(.. | 0.003537 |
| 36 | bef_miRNA*_6 | 0.029594 | 82 | miRNA_U.(( | 0.003896 | 128 | miRNA*_C… | 0.007906 |
| 37 | bef_miRNA*_5 | 0.067404 | 83 | miRNA_U(.( | 0.007869 | 129 | miRNA*_G((( | 0.021414 |
| 38 | bef_miRNA*_4 | 0.089370 | 84 | miRNA_U((. | 0.004541 | 130 | miRNA*_G.(( | 0.002431 |
| 39 | bef_miRNA*_3 | 0.154817 | 85 | miRNA_U..( | 0.052278 | 131 | miRNA*_G(.( | 0.005094 |
| 40 | bef_miRNA*_2 | 0.295159 | 86 | miRNA_U.(. | 0.005083 | 132 | miRNA*_G((. | 0.000452 |
| 41 | bef_miRNA*_1 | 0.300938 | 87 | miRNA_U(.. | 0.038362 | 133 | miRNA*_G..( | 0.005266 |
| 42 | miRNA*_1 | 0.219699 | 88 | miRNA_U… | 0.083804 | 134 | miRNA*_G.(. | 0.001639 |
| 43 | miRNA*_2 | 0.177105 | 89 | miRNA_C((( | 0.030869 | 135 | miRNA*_G(.. | 0.000932 |
| 44 | miRNA*_3 | 0.156106 | 90 | miRNA_C.(( | 0.000000 | 136 | miRNA*_G… | 0.006126 |
| 45 | miRNA*_4 | 0.162910 | 91 | miRNA_C(.( | 0.032063 | 137 | miRNA_5'end | 0.173950 |
| 46 | miRNA*_5 | 0.104964 | 92 | miRNA_C((. | 0.018328 | 138 | miRNA*_5'end | 0.100109 |

(b) The information gain of all 138 features for the 3′ miRNA samples

| Rank | AttrName | IG(c, attr) | Rank | AttrName | IG(c, attr) | Rank | AttrName | IG(c, attr) |
| --- | --- | --- | --- | --- | --- | --- | --- | --- |
| 1 | dis | 1.000000 | 47 | miRNA*_6 | 0.058409 | 93 | miRNA_C..( | 0.016264 |
| 2 | bef_miRNA_6 | 0.093634 | 48 | miRNA*_7 | 0.068116 | 94 | miRNA_C.(. | 0.002884 |
| 3 | bef_miRNA_5 | 0.042059 | 49 | miRNA*_8 | 0.038545 | 95 | miRNA_C(.. | 0.035812 |
| 4 | bef_miRNA_4 | 0.027645 | 50 | miRNA*_9 | 0.021153 | 96 | miRNA_C… | 0.059303 |
| 5 | bef_miRNA_3 | 0.079485 | 51 | miRNA*_10 | 0.015416 | 97 | miRNA_G((( | 0.059992 |
| 6 | bef_miRNA_2 | 0.107534 | 52 | miRNA*_11 | 0.028479 | 98 | miRNA_G.(( | 0.013240 |
| 7 | bef_miRNA_1 | 0.142261 | 53 | miRNA*_12 | 0.035426 | 99 | miRNA_G(.( | 0.012845 |
| 8 | miRNA_1 | 0.415100 | 54 | miRNA*_13 | 0.046686 | 100 | miRNA_G((. | 0.000629 |
| 9 | miRNA_2 | 0.104714 | 55 | miRNA*_14 | 0.067225 | 101 | miRNA_G..( | 0.018624 |
| 10 | miRNA_3 | 0.106573 | 56 | miRNA*_15 | 0.060882 | 102 | miRNA_G.(. | 0.011202 |
| 11 | miRNA_4 | 0.088494 | 57 | miRNA*_16 | 0.055318 | 103 | miRNA_G(.. | 0.014267 |
| 12 | miRNA_5 | 0.052781 | 58 | miRNA*_17 | 0.066825 | 104 | miRNA_G… | 0.035904 |
| 13 | miRNA_6 | 0.042234 | 59 | miRNA*_18 | 0.053781 | 105 | miRNA*_A((( | 0.006308 |
| 14 | miRNA_7 | 0.060406 | 60 | miRNA*_19 | 0.087874 | 106 | miRNA*_A.(( | 0.001278 |
| 15 | miRNA_8 | 0.040500 | 61 | miRNA*_20 | 0.180308 | 107 | miRNA*_A(.( | 0.002708 |
| 16 | miRNA_9 | 0.034058 | 62 | miRNA*_21 | 0.138536 | 108 | miRNA*_A((. | 0.000890 |
| 17 | miRNA_10 | 0.013317 | 63 | miRNA*_22 | 0.119707 | 109 | miRNA*_A..( | 0.002145 |
| 18 | miRNA_11 | 0.013641 | 64 | aft_miRNA*_1 | 0.102586 | 110 | miRNA*_A.(. | 0.000000 |
| 19 | miRNA_12 | 0.037707 | 65 | aft_miRNA*_2 | 0.057072 | 111 | miRNA*_A(.. | 0.001139 |
| 20 | miRNA_13 | 0.042621 | 66 | aft_miRNA*_3 | 0.066437 | 112 | miRNA*_A… | 0.001804 |
| 21 | miRNA_14 | 0.079158 | 67 | aft_miRNA*_4 | 0.089244 | 113 | miRNA*_U((( | 0.002515 |
| 22 | miRNA_15 | 0.061719 | 68 | aft_miRNA*_5 | 0.209931 | 114 | miRNA*_U.(( | 0.002376 |
| 23 | miRNA_16 | 0.091685 | 69 | aft_miRNA*_6 | 0.276058 | 115 | miRNA*_U(.( | 0.002923 |
| 24 | miRNA_17 | 0.106052 | 70 | MFE1 | 0.338455 | 116 | miRNA*_U((. | 0.000334 |
| 25 | miRNA_18 | 0.112559 | 71 | MFE2 | 0.290285 | 117 | miRNA*_U..( | 0.004006 |
| 26 | miRNA_19 | 0.097091 | 72 | MFE3 | 0.158735 | 118 | miRNA*_U.(. | 0.001937 |
| 27 | miRNA_20 | 0.105786 | 73 | miRNA_A((( | 0.065363 | 119 | miRNA*_U(.. | 0.006410 |
| 28 | miRNA_21 | 0.169670 | 74 | miRNA_A.(( | 0.003979 | 120 | miRNA*_U… | 0.003574 |
| 29 | miRNA_22 | 0.186444 | 75 | miRNA_A(.( | 0.034759 | 121 | miRNA*_C((( | 0.001281 |
| 30 | aft_miRNA_1 | 0.149223 | 76 | miRNA_A((. | 0.002796 | 122 | miRNA*_C.(( | 0.003007 |
| 31 | aft_miRNA_2 | 0.096638 | 77 | miRNA_A..( | 0.010517 | 123 | miRNA*_C(.( | 0.001337 |
| 32 | aft_miRNA_3 | 0.085045 | 78 | miRNA_A.(. | 0.001795 | 124 | miRNA*_C((. | 0.005799 |
| 33 | aft_miRNA_4 | 0.053901 | 79 | miRNA_A(.. | 0.016021 | 125 | miRNA*_C..( | 0.003766 |
| 34 | aft_miRNA_5 | 0.041529 | 80 | miRNA_A… | 0.017292 | 126 | miRNA*_C.(. | 0.001380 |
| 35 | aft_miRNA_6 | 0.034978 | 81 | miRNA_U((( | 0.064646 | 127 | miRNA*_C(.. | 0.003384 |
| 36 | bef_miRNA*_6 | 0.068153 | 82 | miRNA_U.(( | 0.003246 | 128 | miRNA*_C… | 0.002533 |
| 37 | bef_miRNA*_5 | 0.090242 | 83 | miRNA_U(.( | 0.005242 | 129 | miRNA*_G((( | 0.004536 |
| 38 | bef_miRNA*_4 | 0.086148 | 84 | miRNA_U((. | 0.003259 | 130 | miRNA*_G.(( | 0.005386 |
| 39 | bef_miRNA*_3 | 0.135579 | 85 | miRNA_U..( | 0.025244 | 131 | miRNA*_G(.( | 0.001841 |
| 40 | bef_miRNA*_2 | 0.168616 | 86 | miRNA_U.(. | 0.005896 | 132 | miRNA*_G((. | 0.000846 |
| 41 | bef_miRNA*_1 | 0.158711 | 87 | miRNA_U(.. | 0.012748 | 133 | miRNA*_G..( | 0.004986 |
| 42 | miRNA*_1 | 0.097427 | 88 | miRNA_U… | 0.052932 | 134 | miRNA*_G.(. | 0.001188 |
| 43 | miRNA*_2 | 0.090714 | 89 | miRNA_C((( | 0.036791 | 135 | miRNA*_G(.. | 0.005557 |
| 44 | miRNA*_3 | 0.106627 | 90 | miRNA_C.(( | 0.022137 | 136 | miRNA*_G… | 0.002943 |
| 45 | miRNA*_4 | 0.102434 | 91 | miRNA_C(.( | 0.016463 | 137 | miRNA_5'end | 0.157376 |
| 46 | miRNA*_5 | 0.084488 | 92 | miRNA_C((. | 0.006283 | 138 | miRNA*_5'end | 0.122820 |

(c) The information gain of all 138 features for the combined training dataset, including both 5′ and 3′ miRNA samples

| Rank | AttrName | IG(c, attr) | Rank | AttrName | IG(c, attr) | Rank | AttrName | IG(c, attr) |
| --- | --- | --- | --- | --- | --- | --- | --- | --- |
| 1 | dis | 1.000000 | 47 | miRNA*_6 | 0.098516 | 93 | miRNA_C..( | 0.030188 |
| 2 | bef_miRNA_6 | 0.087131 | 48 | miRNA*_7 | 0.093553 | 94 | miRNA_C.(. | 0.008264 |
| 3 | bef_miRNA_5 | 0.036076 | 49 | miRNA*_8 | 0.047408 | 95 | miRNA_C(.. | 0.044243 |
| 4 | bef_miRNA_4 | 0.034690 | 50 | miRNA*_9 | 0.036949 | 96 | miRNA_C… | 0.079702 |
| 5 | bef_miRNA_3 | 0.067636 | 51 | miRNA*_10 | 0.027617 | 97 | miRNA_G((( | 0.151489 |
| 6 | bef_miRNA_2 | 0.108302 | 52 | miRNA*_11 | 0.035898 | 98 | miRNA_G.(( | 0.001769 |
| 7 | bef_miRNA_1 | 0.133346 | 53 | miRNA*_12 | 0.062580 | 99 | miRNA_G(.( | 0.022549 |
| 8 | miRNA_1 | 0.789216 | 54 | miRNA*_13 | 0.079488 | 100 | miRNA_G((. | 0.000845 |
| 9 | miRNA_2 | 0.137712 | 55 | miRNA*_14 | 0.129497 | 101 | miRNA_G..( | 0.018146 |
| 10 | miRNA_3 | 0.147577 | 56 | miRNA*_15 | 0.121085 | 102 | miRNA_G.(. | 0.015628 |
| 11 | miRNA_4 | 0.150282 | 57 | miRNA*_16 | 0.102916 | 103 | miRNA_G(.. | 0.009819 |
| 12 | miRNA_5 | 0.095660 | 58 | miRNA*_17 | 0.115490 | 104 | miRNA_G… | 0.046316 |
| 13 | miRNA_6 | 0.086529 | 59 | miRNA*_18 | 0.077938 | 105 | miRNA*_A((( | 0.007350 |
| 14 | miRNA_7 | 0.123406 | 60 | miRNA*_19 | 0.128055 | 106 | miRNA*_A.(( | 0.001599 |
| 15 | miRNA_8 | 0.070077 | 61 | miRNA*_20 | 0.272366 | 107 | miRNA*_A(.( | 0.003325 |
| 16 | miRNA_9 | 0.068828 | 62 | miRNA*_21 | 0.120604 | 108 | miRNA*_A((. | 0.000370 |
| 17 | miRNA_10 | 0.015909 | 63 | miRNA*_22 | 0.119963 | 109 | miRNA*_A..( | 0.002488 |
| 18 | miRNA_11 | 0.030166 | 64 | aft_miRNA*_1 | 0.074770 | 110 | miRNA*_A.(. | 0.001106 |
| 19 | miRNA_12 | 0.073708 | 65 | aft_miRNA*_2 | 0.045220 | 111 | miRNA*_A(.. | 0.003605 |
| 20 | miRNA_13 | 0.061846 | 66 | aft_miRNA*_3 | 0.063302 | 112 | miRNA*_A… | 0.004655 |
| 21 | miRNA_14 | 0.109783 | 67 | aft_miRNA*_4 | 0.072861 | 113 | miRNA*_U((( | 0.006370 |
| 22 | miRNA_15 | 0.104560 | 68 | aft_miRNA*_5 | 0.177566 | 114 | miRNA*_U.(( | 0.001602 |
| 23 | miRNA_16 | 0.138205 | 69 | aft_miRNA*_6 | 0.209295 | 115 | miRNA*_U(.( | 0.004474 |
| 24 | miRNA_17 | 0.168535 | 70 | MFE1 | 0.577517 | 116 | miRNA*_U((. | 0.001310 |
| 25 | miRNA_18 | 0.170835 | 71 | MFE2 | 0.443980 | 117 | miRNA*_U..( | 0.005577 |
| 26 | miRNA_19 | 0.181860 | 72 | MFE3 | 0.256573 | 118 | miRNA*_U.(. | 0.002533 |
| 27 | miRNA_20 | 0.208319 | 73 | miRNA_A((( | 0.088863 | 119 | miRNA*_U(.. | 0.007421 |
| 28 | miRNA_21 | 0.294693 | 74 | miRNA_A.(( | 0.010006 | 120 | miRNA*_U… | 0.007670 |
| 29 | miRNA_22 | 0.293491 | 75 | miRNA_A(.( | 0.049161 | 121 | miRNA*_C((( | 0.003569 |
| 30 | aft_miRNA_1 | 0.200173 | 76 | miRNA_A((. | 0.006345 | 122 | miRNA*_C.(( | 0.001558 |
| 31 | aft_miRNA_2 | 0.139260 | 77 | miRNA_A..( | 0.011316 | 123 | miRNA*_C(.( | 0.002571 |
| 32 | aft_miRNA_3 | 0.118057 | 78 | miRNA_A.(. | 0.003478 | 124 | miRNA*_C((. | 0.006859 |
| 33 | aft_miRNA_4 | 0.045330 | 79 | miRNA_A(.. | 0.039740 | 125 | miRNA*_C..( | 0.005702 |
| 34 | aft_miRNA_5 | 0.020331 | 80 | miRNA_A… | 0.029719 | 126 | miRNA*_C.(. | 0.004850 |
| 35 | aft_miRNA_6 | 0.025063 | 81 | miRNA_U((( | 0.118453 | 127 | miRNA*_C(.. | 0.002995 |
| 36 | bef_miRNA*_6 | 0.053818 | 82 | miRNA_U.(( | 0.004094 | 128 | miRNA*_C… | 0.007311 |
| 37 | bef_miRNA*_5 | 0.100710 | 83 | miRNA_U(.( | 0.009872 | 129 | miRNA*_G((( | 0.014912 |
| 38 | bef_miRNA*_4 | 0.111635 | 84 | miRNA_U((. | 0.005098 | 130 | miRNA*_G.(( | 0.002065 |
| 39 | bef_miRNA*_3 | 0.179885 | 85 | miRNA_U..( | 0.046357 | 131 | miRNA*_G(.( | 0.002637 |
| 40 | bef_miRNA*_2 | 0.277593 | 86 | miRNA_U.(. | 0.007720 | 132 | miRNA*_G((. | 0.001516 |
| 41 | bef_miRNA*_1 | 0.293118 | 87 | miRNA_U(.. | 0.019580 | 133 | miRNA*_G..( | 0.004270 |
| 42 | miRNA*_1 | 0.219999 | 88 | miRNA_U… | 0.079902 | 134 | miRNA*_G.(. | 0.001426 |
| 43 | miRNA*_2 | 0.174354 | 89 | miRNA_C((( | 0.028094 | 135 | miRNA*_G(.. | 0.001817 |
| 44 | miRNA*_3 | 0.158600 | 90 | miRNA_C.(( | 0.000000 | 136 | miRNA*_G… | 0.005570 |
| 45 | miRNA*_4 | 0.162212 | 91 | miRNA_C(.( | 0.025376 | 137 | miRNA_5'end | 0.245784 |
| 46 | miRNA*_5 | 0.122546 | 92 | miRNA_C((. | 0.012200 | 138 | miRNA*_5'end | 0.183690 |
